# Transcriptomic analysis of gene expression of *Verticillium dahliae* upon treatment of the cotton root exudates

**DOI:** 10.1186/s12864-020-6448-9

**Published:** 2020-02-12

**Authors:** Xinyu Zhang, Wenhan Cheng, Zhidi Feng, Qianhao Zhu, Yuqiang Sun, Yanjun Li, Jie Sun

**Affiliations:** 1 The Key Laboratory of Oasis Eco-agriculture, Shihezi University College of Agriculture, Shihezi city, Xinjiang, 832003 China; 2 CSIRO Agriculture and Food, GPO Box 1700, Canberra, 2601 Australia; 3 Zhejiang Sci-Tech University College of Life Sciences, Zhejiang, 310016 Hangzhou China

**Keywords:** Root exudates, Transcriptome, *Verticillium dahliae*, Hydrolase activity, hydrolyzing O-glycosyl compounds hydrolase

## Abstract

**Background:**

Cotton Verticillium wilt is one of the most devastating diseases for cotton production in the world. Although this diseases have been widely studied at the molecular level from pathogens, the molecular basis of *V. dahliae* interacted with cotton has not been well examined.

**Results:**

In this study, RNA-seq analysis was carried out on *V. dahliae* samples cultured by different root exudates from three cotton cultivars (a susceptible upland cotton cultivar, a tolerant upland cotton cultivar and a resistant island cotton cultivar) and water for 0 h, 6 h, 12 h, 24 h and 48 h. Statistical analysis of differentially expressed genes revealed that *V. dahliae* responded to all kinds of root exudates but more strongly to susceptible cultivar than to tolerant and resistant cultivars. Go analysis indicated that ‘hydrolase activity, hydrolyzing O-glycosyl compounds’ related genes were highly enriched in *V. dahliae* cultured by root exudates from susceptible cotton at early stage of interaction, suggesting genes related to this term were closely related to the pathogenicity of *V. dahliae*. Additionally, ‘transmembrane transport’, ‘coenzyme binding’, ‘NADP binding’, ‘cofactor binding’, ‘oxidoreductase activity’, ‘flavin adenine dinucleotide binding’, ‘extracellular region’ were commonly enriched in *V. dahliae* cultured by all kinds of root exudates at early stage of interaction (6 h and 12 h), suggesting that genes related to these terms were required for the initial steps of the roots infections.

**Conclusions:**

Based on the GO analysis results, the early stage of interaction (6 h and 12 h) were considered as the critical stage of *V. dahliae*-cotton interaction. Comparative transcriptomic analysis detected that 31 candidate genes response to root exudates from cotton cultivars with different level of *V. dahliae* resistance, 68 response to only susceptible cotton cultivar, and 26 genes required for development of *V. dahliae*. Collectively, these expression data have advanced our understanding of key molecular events in the *V. dahliae* interacted with cotton, and provided a framework for further functional studies of candidate genes to develop better control strategies for the cotton wilt disease.

## Background

*Verticillium dahliae* (*V. dahliae*), a fungal pathogen causing Verticillium wilt, is extremely persistent in the soil and has a broad host range [1, 2]. Microsclerotia of *V. dahliae* overcome the mycostatic activity of the soil and germinate towards roots in the presence of root exudates [3]. The hyphae enter host plants by formation of an infection structure, known as hyphopodium, to develop a penetration peg to pierce root epidermal cells [4]. They enter and clog the xylem vessels, resulting in leaf curl, necrosis, defoliation, vascular tissue wilt, and discoloration [5]. During its life cycle, cotton is continuously threatened by *V. dahliae*. More than half of the cotton fields in China are affected by *V. dahliae* and can lead to 30–50% reduction in yield, and even totally wipe out the crop. Verticillium wilt is one of the most severe cotton diseases not only in China but also in other countries. Outbreak of the disease causes substantial economic loss due to significant reduction in fiber yield and quality.

To combat the challenge of *V. dahliae,* resistance cotton has evolved multiple layers of defense mechanisms, including tissue composition, physiological and biochemical resistance, during the long time period of coexistence and arm race [6–10]. In recent years, with the application of genomics, transcriptomics and proteomics, great progress has been made in understanding the molecular mechanism underlying cotton’s resistance against *V. dahliae*, and a number of genes related to *V. dahliae* resistance have been identified [11–16]. On the other hand, in view of the co-evolving relationship between cotton and *V. dahliae*, it is also of vital importance to study the molecular mechanisms determining the pathogenicity of *V. dahliae*. With the completion of genome sequencing of *V. dahliae* and the development of bioinformatics tools, genomic and transcriptomic sequence information of *V. dahliae* provide us opportunity for better understanding the pathogenicity of *V. dahliae*. Analyses of *V. dahliae* transcriptomes during microsclerotia formation and early infection stage have given us a snapshot of the genes important for development, microsclerotia formation and infection of *V. dahliae* [17–20]. For instance, *VdPKAC1*, *VMK1*, *VdMsb*, *VdGARP1*, *VDH1*, *Vayg1* and *VGB* were found to be involved in the microsclerotia formation and pathogenic process of *V. dahliae* [3, 21–26]; *VdSNF1* and *VdSSP1* are related to cell wall degradation [27, 28]; *VdNEP*, *VdpevD1, VdNLP1* and *VdNLP2* encode effector proteins are involved in the pathogenic reaction [29–32]; *VdFTF1*, *Vta2* and *VdSge1* encode transcriptional factors regulating pathogenic genes [33–35]. However, due to the complexity of the pathogenic molecular mechanism of *V. dahliae*, we still know little about the role of these genes in the interaction between *V. dahliae* and cotton.

Successful pathogens must be able to recognize and overcome host-plant defense responses [36]. *V. dahliae* invades cotton through the root system [4, 37], therefore, the biological effect of the root exudates is expected to be crucial for successful infection of *V. dahliae*. Not surprisingly, root exudates have been found to be closely related to plant resistance [38, 39]. The root exudates of cotton are rich in amino acids and sugars. Compared with the root exudates from the susceptible cotton cultivars, the root exudates from resistant cotton cultivars lacked aspartic acid, threonine, glutamic acid, alanine, isoleucine, leucine, phenylalanine, lysine and proline, but contained arginine that was absent in the susceptible cottons. No significant difference of saccharide was found in the root exudates between the susceptible and resistant cultivars, but the root exudates of the susceptible cultivars had a much higher concentrations of glucose, fructose and sucrose than that of the resistant ones [40]. Root exudates from the resistant and susceptible cottons inhibited and promoted the growth of *V. dahliae*, respectively [40–42]. However, we know nothing about the molecular basis behind this observation.

In this study, we investigated the effects of root exudates from cotton cultivars susceptible, tolerant or resistant to *V. dahliae* on the development of the pathogen and performed a time course expression analysis of *V. dahliae* genes using RNA-seq to (1) compare transcriptomic profiles of *V. dahliae* in response to root exudates from cottons with different level of *V. dahliae* resistance, (2) identify biological processes in *V. dahliae* affected by different root exudates based on analysis of Gene Ontology (GO) terms of the differentially expressed genes, and (3) identify genes involved in the initial steps of roots infection and likely in pathogenesis of *V. dahliae*. We expect that identification of pathogenic genes in *V. dahliae* would provide us clues to develop novel strategies for breeding novel cotton germplasm resistant to *V. dahliae* and/or effective crop management schemes to minimize the infection of *V. dahliae.*

## Methods

### Cotton cultivars and *V. dahliae* strain

Two Upland cotton (*G. hirsutum* L.) cultivars Xinluzao 8 (X) and Zhongzhimian 2 (Z), and one Sea island (*G. barbadense* L.) cultivar Hai7124 (H) used in this study were collected from the Institute of Cotton Research of Chinese Academy of Agricultural Sciences (Anyang, China) and Shihezi Academy of Agricultural Sciences (Shihezi, China). The 3 cotton cultivars were authorized for only scientific research purpose, and were deposited in the original institutes and College of Agriculture in Shihezi University. The highly virulent *V. dahliae* strain*,* V991, was provided and confirmed by the Institute of Cotton Research of Chinese Academy of Agricultural Sciences (Anyang, China). The growth conditions of the cotton cultivars, the preparation of *V.dahliae* spore suspensions for infection assays and determination of Disease Index after inoculation were described previously [43, 44].

### Collection of root exudates

Xinluzao 8, Zhongzhimian 2 and Hai7124 are susceptible, tolerant and resistant to *V. dahliae*, respectively. Cotton seeds were surface sterilized by immersion in 1% (w/v) NaClO and rinsed three times with sterile distilled water. After germination in petri dish, the seeds were sown in sand that were treated by soaking in dilute suphuric acid and sterilized by high temperature. For each cultivar 18 germinated seeds were evenly planted in 2 pots and were grown in a greenhouse with a photoperiod of 16 h light/8 h darkness at 28 °C. The cotton seedlings were fed with Hoagland nutrient solution every 3 days (3d). After 45d, the plants were removed from sand, and the sand was immersed with 2 L distilled water to sufficiently dissolve root exudates. The water solution was then filtered with a bacterial filter (0.22 μm in diameter) and concentrated to 0.5 L in a freeze dryer.

### *V. dahliae* strain culture

*V. dahliae* strain*,* V991, was maintained in 20% glycerol at − 80 °C at the Key Laboratory of Oasis Eco-agriculture in Shihezi University. The stored conidia of V991 were incubated on a potato–dextrose agar plate for 1 week and then inoculated into Czapek broth for 5d at 25 °C 180 rpm under dark donditions. The fresh conidia and spores were then collected to be used in the root exudate treatment experiments. For each cultivar, 0.5 g of V991 conidia and spores were suspended in 5 mL of root exudates. After cultured for 6, 12, 24 or 48 h at 25 °C 220 rpm in 10 mL centrifugal tubes, V991 conidia and spores (Vd-X-6, Vd-X-12, Vd-X-24, Vd-X-48, Vd-Z-6, Vd-Z-12, Vd-Z-24, Vd-Z-48, Vd-H-6, Vd-H-12, Vd-H-24 and Vd-H-48) were collected for RNA extraction. The same amount of V991 conidia and spores suspended in water and cultured for 0, 6, 12, 24 or 48 h were done in parallel (Vd-0, Vd-W-6, Vd-W-12, Vd-W-24 and Vd-W-48). Each time point had two biological replicates. In total, 34 samples were collected and used in RNA-seq.

### RNA extraction

Total RNA of *V. dahliae* was isolated using the RNA simple total RNA kit (Tiagen, Beijing, China) according to the manufacturer’s protocol. All RNA samples were treated with RNase-free DNase I. Degradation and contamination of RNA were assessed by using agarose gel electrophoresis. The RNA purity and integrity were determined by a NanoDrop® 2000 spectrophotometer (Thermo Scientific, Wilmington, DE, USA) and an Agilent 2100 bioanalyzer (Agilent Technologies, Santa Clara, CA, USA). The RNA concentration was measured by a Qubit® 2.0 Fluorometer (Thermo Scientific, Wilmington, DE, USA). High quality RNA samples were chosen for RNA-Seq analyses.

### RNA-Seq library construction and sequencing

RNA-Seq library preparation and sequencing were performed at Novogene Bioinformatics Technology Co., Ltd. (Beijing, China) using the standard Illumina protocols. Briefly, mRNAs were enriched from 1.5 μg total RNA by using magnetic beads with Oligo (dT), and then fragmented by adding fragmentation buffer. The short fragments were used as templates to synthesize the first stranded cDNAs with random hexamers. Double-stranded cDNAs were then synthesized by using DNA Polymerase I and RNase H and purified with AMPure XP beads. The purified double-stranded cDNAs was then end repaired, added A tail and ligated with sequencing adapters. The products were enriched with PCR to create the final cDNA libraries. Finally, the library was sequenced on the Illumina Hiseq™ 4000 platform (Illumina, San Diego, CA, USA, 2010).

### RNA-Seq data analysis and identification of differentially expressed genes

Raw reads were pre-processed by removing low quality sequences and adaptor using Trimmomatic [45]. The Q30 values, GC content, and sequence duplication levels were calculated for the clean data. All downstream analysis used the clean data with high quality. The resulting high-quality clean reads were then aligned to *V. dahliae* sequence from genome database (http//www.broadinstitute.org/annotation/genome/*Verticillium* *dahliae*/Blast.html) using the HISAT software [46]. Following alignment, raw read counts for each *V. dahliae* gene were generated and normalized to FPKM (fragments per kilobase of exon model per million mapped fragments) [47]. The expression level of each gene was analyzed using the union model implemented in the HTSeq software [48]. Differentially expressed genes (DEGs) were identified by using the DEGseq software with the following criteria: a fold change> 2.0 and an adjusted *p* value<0.05 [49]. Gene ontology (GO) term enrichment analysis of DEGs was performed based on the Wallenius non-central hyper-geometric distribution using the GOseq software [50].

### qRT-PCR confirmation of differentially expressed genes

Total RNA from *V. dahliae* was isolated as mentioned above. One microgram of total RNA was used for first-strand cDNA synthesis with the M-MLV reverse transcriptase (TaKaRa, Dalian) according to the manufacturer’s instructions. The cDNAs were then used as templates for quantitative real-time PCR (qRT-PCR) experiments. The gene specific primers used in qRT-PCR are listed in Table 1, and the *V. dahliae tubulin* gene was used as an internal control. The qRT-PCR assays were performed with SYBR Premix Ex Taq (TaKaRa) on a LightCycler 480 system (Roche, USA). All reactions were measured in triplicate. The relative expression ratio of each gene was calculated from the cycle threshold (CT) values using the 2^-ΔΔCT^ method.

**Table 1:** Primers used in qRT-PCR to validate RNA-seq data

Accession no.	Gene description	Primiers
VDAG_10074	tubulin	5' TCCACCTTCGTCGGTAACTC 3'
		5' GCCTCCTCCTCGTACTCCTC 3'
VDAG_01193	high-affinity nicotinic acid transporter	5' GTGCCATCTCCGGCTTCATC 3'
		5' TTGCGTTGTCACCCTTCTCG 3'
VDAG_01866	xylosidase/arabinosidase	5' CAGCTCCGTGCTCAATGTGCC 3'
		5' TCCAACTGAGATGCCCGCCTT 3'
VDAG_03038	periplasmic trehalase	5' GGCAACAACCTCACTCGC 3'
		5' GCACTACGGCTACCAAACTTCT 3'
VDAG_03526	alpha-glucuronidase	5' GTGACGGCGGACAACTCTAC 3'
		5' TGCACGCCCTTGAATGTAAT 3'
VDAG_04513	hexose transporter protein	5' TCAACATTGCCATCCAGGTC 3'
		5' CGAAGCACAGCTCGAAGAAG 3'
VDAG_07563	sugar transporter STL1	5' AGTGCCCGTCGTCTACTTCTT 3'
		5' GTTCTTGCCGTAACGCCTC 3'
VDAG_08286	alpha-glucosides permease MPH2/3	5' GTATCGGCCAGACCAACCA 3'
		5' CATCGCCACCATTTAACCC 3'
VDAG_09088	MFS transporter	5' AGGAGAAGAAGGCCGTCGTG 3'
		5' CCGTAAAGATTGCCGTGGTC 3'

## Results

### Identification of cotton resistance to *V. dahliae* infection

**Fig. 1:**
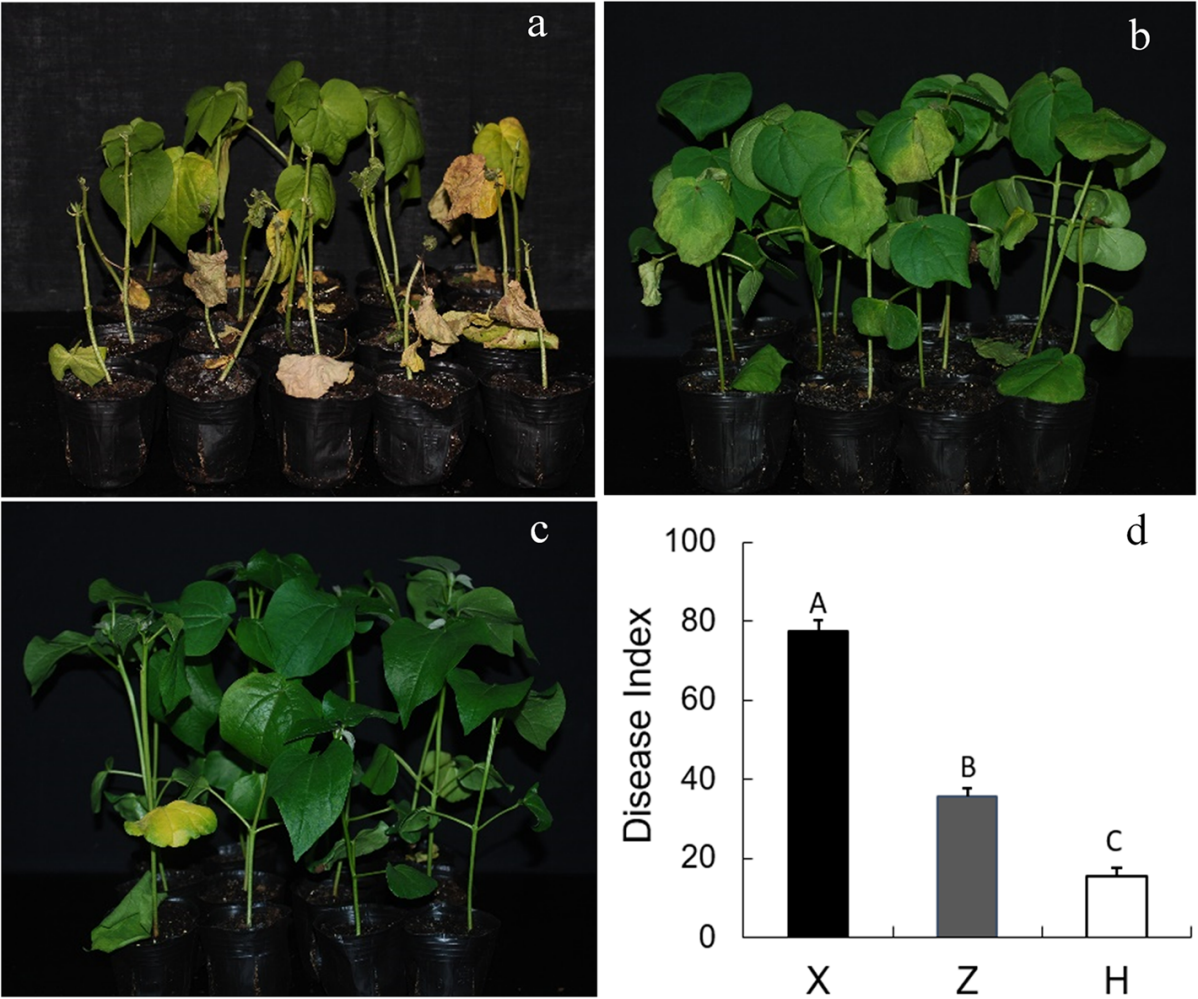
Disease symptoms of V991 infection on Xinluzao 8, Zhongzhimian 2 and Hai 7124. The photograph was taken at 20 days post-inoculation. **a**. Disease symptoms of V991 infection on Xinluzao 8. **b**. Disease symptoms of V991 infection on Zhongzhimian 2. **c**. Disease symptoms of V991 infection on Hai 7124. **d**. Disease index of V991 on Xinluzao 8 (X), Zhongzhimian 2 (Z) and Hai 7124 (H). Different capital letters indicate significant differences (*p* < 0.01) using Duncan’s multiple range test

In this study, three cotton cultivars with different level of *V. dahliae* resistance were selected for collection of root exudates. As can be seen from the Fig. 1, severe leaf wilt disease symptoms and premature defoliation were visually apparent for Xinluzao 8, moderate but typical leaf wilt symptoms were observed in Zhongzhimian 2, whereas only weak wilt disease symptoms were observed in Hai 7124 at 20 days post inoculation. Compared with Xinluzao 8, Zhongzhimian 2 and Hai 7124 exhibited various degrees of resistance to V991 infection with significantly reduced Disease Index in inoculated seedlings (Fig. 1d). According to our results about identification of cotton resistance to *V. dahliae* and previous reports [43, 51], Xinluzao 8, Zhongzhimian 2 and Hai7124 were used as cultivars of susceptible, tolerant and resistant to *V. dahliae*, respectively.

### RNA-seq and transcriptome profiles of *V. dahliae*

To explore the transcriptomic profiling of V991 interacting with root exudates from cotton cultivars with different level of *V. dahliae* resistance, we generated a total of 34 RNA-seq datasets, 24 from *V. dahliae* treated by cotton root exudates (Vd-X-6, Vd-X-12, Vd-X-24, Vd-X-48, Vd-Z-6, Vd-Z-12, Vd-Z-24, Vd-Z-48, Vd-H-6, Vd-H-12, Vd-H-24 and Vd-H-48, each with two replicates), 8 from *V. dahliae* treated by water (Vd-W-6, Vd-W-12, Vd-W-24 and Vd-W-48, each with two replicates) and 2 from untreated *V. dahliae,* i.e. Vd-0.

**Fig. 2:**
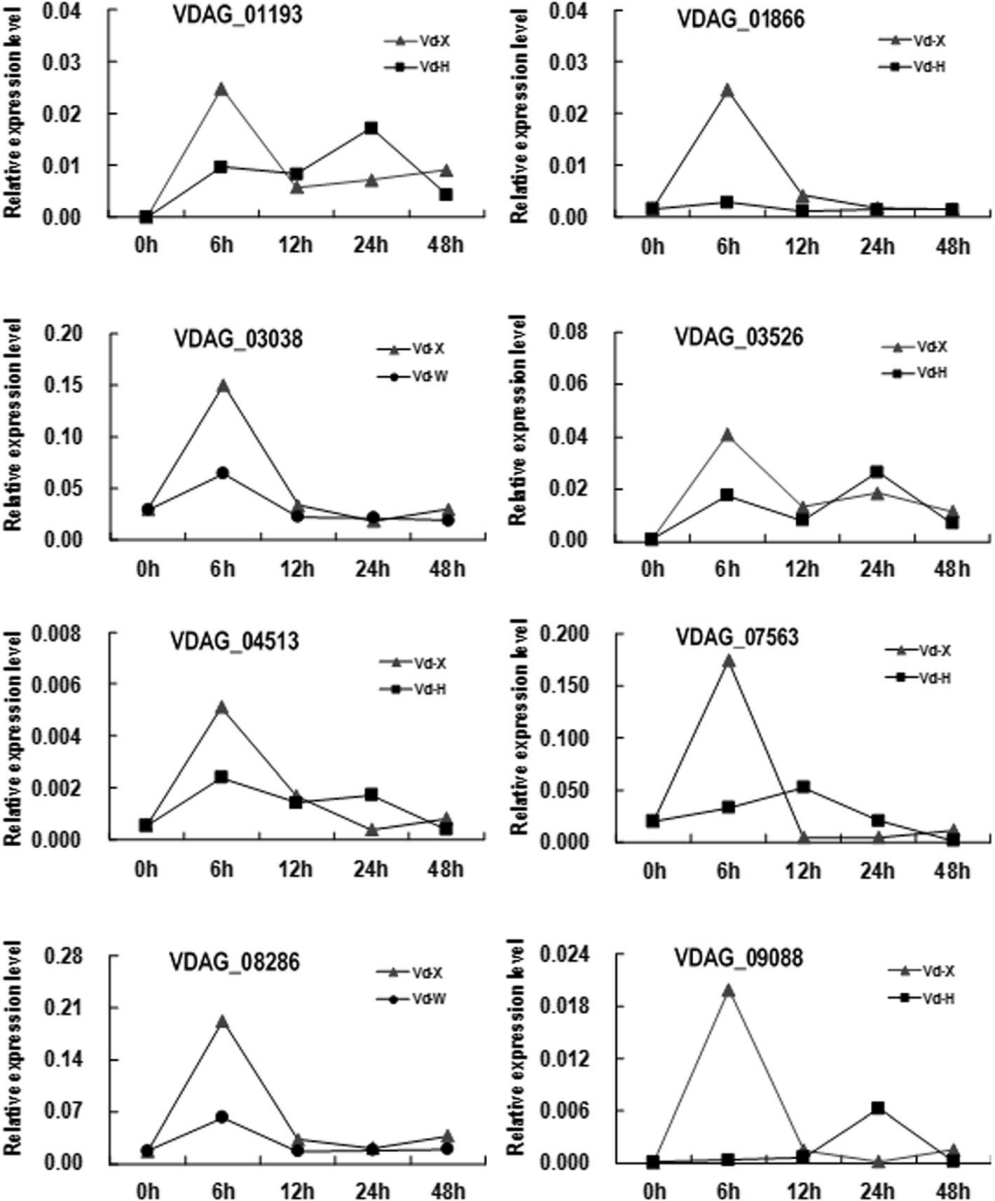
The qRT-PCR analyses of the expression of 8 DEGs selected from all DEGs. The 8 DEGs included VDAG_03038 encoding periplasmic trehalase, VDAG_03526 encoding Alpha-glucuronidase, VDAG_04513 encoding hexose transporter protein, VDAG_05015 encoding beta-galactosidase, VDAG_07563 encoding sugar transporter STL1, VDAG_08212 encoding lactose permease, VDAG_08286 encoding alpha-glucosides permease MPH2/3, VDAG_09088 encoding MFS transporter. The *V. dahliae tubulin* gene (VDAG_10074) was used an internal control. All reactions were measured in triplicate. The expression ratio of the gene was calculated from cycle threshold (CT) values using the 2^-ΔΔCT^ method

An overview of the sequencing results is outlined in Table 2. After discarding the low-quality reads, the total number of clean reads per library ranged from 13 to 22 million, and clean bases ranged from 1.97 to 3.22 Gb. Between 11,657,068 and 19,529,825 of these reads were uniquely mapped to the *V. dahliae* reference genome. The genic distribution of the uniquely mapped reads indicated that most reads (>88.2%) were mapped to exons, and the others were distributed between introns (0.2–0.3%) and intergenic regions (6.7–11.6%) (Additional file 3: Table S1). The Pearson’s correlation coefficients (R^2^) of FPKM distribution between the two biological replicates for each sample were high in each treatment (*R*^2^ = 0.945–0.987, *p*<0.001), indicating a good level of reproducibility of the RNA-seq data (Additional file 1: Figure S1). The RNA-seq results were also confirmed to be reliable by qRT-PCR using 8 randomly selected genes (Table 1, Fig. 2) (Additional file 2: Figure S2). For example, the expression levels of these genes peaked at 6 h in Vd-X, but showed no obvious change in Vd-H and Vd-W.

**Table 2:** Summary of RNA-seq reads generated in the study

Sample name	Raw reads	Clean reads	Clean bases	Error rate (%)	Q20 (%)	Q30 (%)	GC content (%)
Vd-X-6a	20,755,066	19,829,720	2.97G	0.03	94.52	86.95	58.14
Vd-X-6b	20,008,568	19,162,740	2.87G	0.03	95.13	88.19	58.81
Vd-X-12a	20,745,450	19,871,376	2.98G	0.03	94.57	87.09	57.72
Vd-X-12b	17,752,478	15,961,608	2.39G	0.02	97.07	91.35	58.61
Vd-X-24a	18,562,576	17,673,746	2.65G	0.03	94.78	87.43	58.56
Vd-X-24b	18,713,224	17,855,894	2.68G	0.03	94.55	86.95	58.54
Vd-X-48a	22,863,130	21,469,914	3.22G	0.03	94.65	87.59	51.86
Vd-X-48b	21,450,220	20,335,092	3.05G	0.03	94.82	87.74	53.99
Vd-Z-6a	19,384,506	18,572,808	2.79G	0.03	94.54	86.96	58.28
Vd-Z-6b	18,947,746	16,766,560	2.51G	0.02	95.94	89.19	58.19
Vd-Z-12a	19,116,156	16,262,760	2.44G	0.02	96.47	90.18	58.60
Vd-Z-12b	15,846,552	13,820,768	2.07G	0.02	97.05	90.95	56.33
Vd-Z-24a	22,527,850	19,634,838	2.95G	0.02	97.92	93.16	58.10
Vd-Z-24b	22,678,986	19,618,638	2.94G	0.02	97.87	93.04	57.99
Vd-Z-48a	18,786,644	18,043,658	2.71G	0.02	94.62	87.75	51.51
Vd-Z-48b	16,083,890	15,364,870	2.3G	0.02	94.84	88.07	53.31
Vd-H-6a	17,277,272	15,290,714	2.29G	0.03	96.78	90.34	56.08
Vd-H-6b	23,964,812	21,120,448	3.17G	0.02	97.89	93.15	58.23
Vd-H-12a	16,150,508	13,729,988	2.06G	0.02	97.11	90.87	58.16
Vd-H-12b	22,302,818	19,253,012	2.89G	0.02	97.81	92.96	56.86
Vd-H-24a	14,972,868	13,927,336	2.09G	0.03	96.72	90.13	57.15
Vd-H-24b	14,514,160	13,125,772	1.97G	0.03	96.76	90.26	56.60
Vd-H-48a	18,826,776	16,337,922	2.45G	0.02	96.20	89.63	46.23
Vd-H-48b	17,007,508	14,591,964	2.19G	0.02	95.70	89.13	53.93
Vd-W-6a	15,061,222	13,654,598	2.05G	0.03	96.88	90.43	57.86
Vd-W-6b	22,050,470	21,031,720	3.15G	0.02	96.27	90.86	56.02
Vd-W-12a	22,264,268	21,134,386	3.17G	0.02	96.08	90.46	55.72
Vd-W-12b	20,529,690	19,622,372	2.94G	0.02	95.78	89.43	57.29
Vd-W-24a	15,761,394	15,360,174	2.3G	0.02	94.86	88.07	54.29
Vd-W-24b	23,275,930	22,685,328	3.4G	0.02	95.71	89.64	58.51
Vd-W-48a	18,328,720	17,868,854	2.68G	0.02	94.97	88.46	48.13
Vd-W-48b	22,437,742	21,421,106	3.21G	0.03	94.41	87.08	57.12
Vd-0a (CKa)	16,237,488	15,825,126	2.37G	0.02	95.37	88.77	58.51
Vd-0b (CKb)	14,193,496	13,833,776	2.08G	0.02	95.30	88.54	58.72

**Fig. 3:**
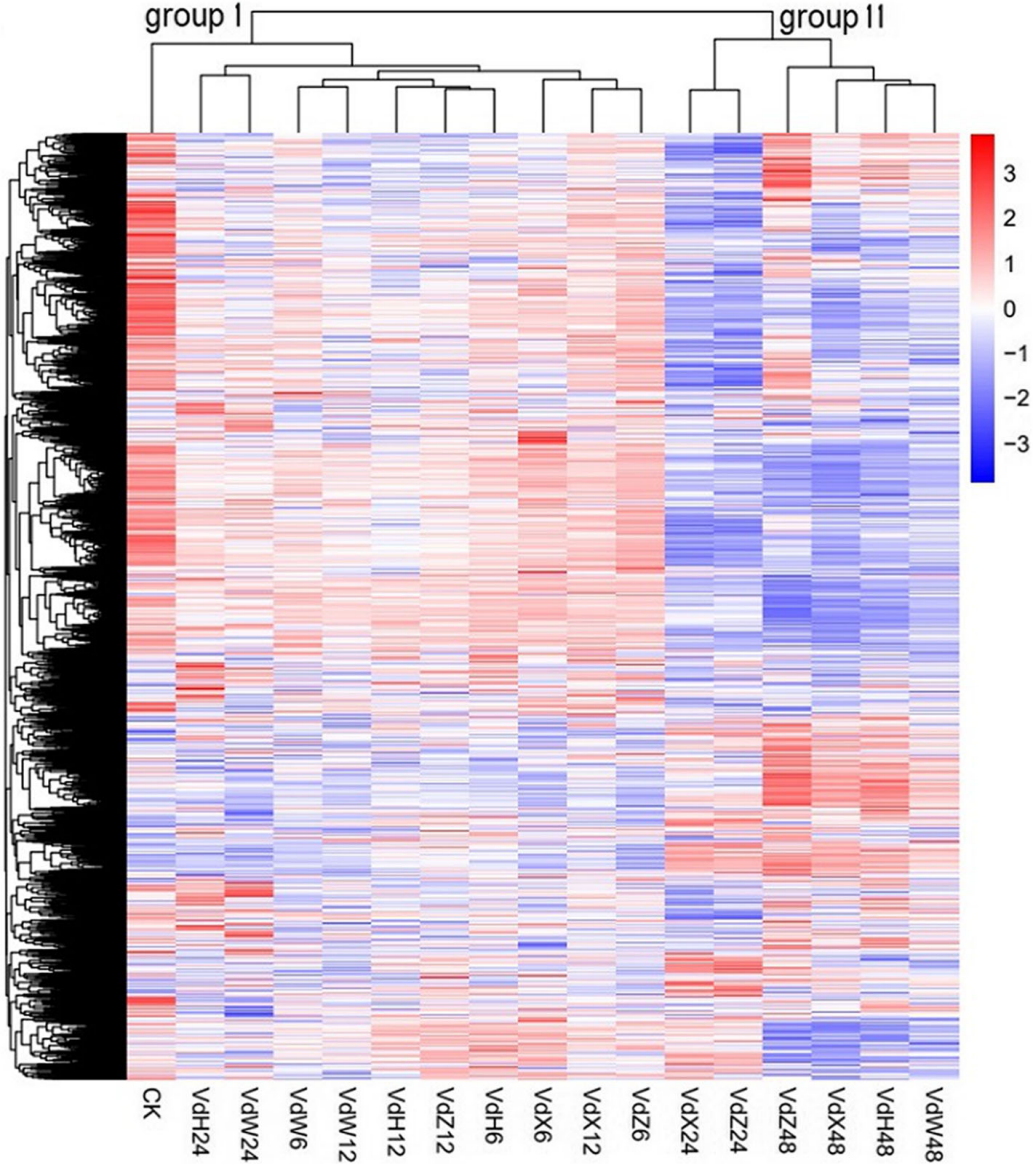
Hierarchical clustering of samples was performed using FPKM values of all genes identified in *V. dahliae*. The log10 (FPKM+ 1) values were normalized and clustered. Red and blue bands represent high and low gene expression genes, respectively. The color ranges from red to blue, indicating that log10 (FPKM + 1) is from large to small

Based on hierarchical clustering using the FPKM values of all genes, it was found that the 17 samples were classified into two groups (Fig. 3). Group I contained all the Vd-6 (Vd-X-6, Vd-Z-6, Vd-H-6 and Vd-W-6) and Vd-12 (Vd-X-12, Vd-Z-12, Vd-H-12 and Vd-W-12) samples as well as Vd-H-24 and Vd-W-24. The expression profiles of these 10 samples were close to that of Vd-0 (CK), which was also clustered in group I. Group II contained all the four Vd-48 (Vd-X-48, Vd-Z-48, Vd-H-48 and Vd-W-48) samples and two Vd-24 (Vd-X-24 and Vd-Z-24) samples. The clustering tree indicated that the gene expression patterns of the two early time points (Vd-6 and Vd-12) were very similar but clearly different from that of the latest time point (Vd-48). The four Vd-24 samples were clustered into the two groups, but were distinct from other samples in the same group by forming a sub-group, suggesting that 24 h could be a transition point regarding the effect of root exudates on the growth of *V. dahliae*.

### Identification of differentially expressed genes (DEGs)

DEGs would offer insights into the metabolic and regulatory changes in *V. dahliae* when interacting with root exudates from cottons with different *V. dahliae* resistance, we thus identified DEGs (*p*<0.05, fold change >2.0) in each interaction using Vd-0 (CK) as a control. Regarding the treatments (root exudates or water), the largest number of DEGs was found in Vd-X vs CK (4602), followed by Vd-Z vs CK (3896), Vd-H vs CK (3227), and Vd-W vs CK (2392) (Table 3), suggesting that *V. dahliae* responded to all kind treatments, but responded more strongly to root exudates from the susceptible cultivar (X) than to those from the tolerant (Z) and resistant cultivars (H). Regarding the effect of treated time, the general trend for Vd-X vs CK, Vd-H vs CK and Vd-W vs CK was that the number of DEGs increased with the increased time of treatment, but for Vd-Z vs CK, there were more DEGs at 24 h than other time points. In all three treatments with root exudates, it seemed there were more up-regulated DEGs than down-regulated DEGs at 6 h, but more down-regulated DEGs than up-regulated ones at other time points (12 h, 24 h and 48 h) (Table 3).

**Table 3:** Statistics of differentially expressed genes of samples vs Vd-0 (CK)

Comparisons		Number of DEGs			
		Up-regulated	Down-regulated	Total	
Vd-X vs CK	Vd-X-6 h vs CK	209	93	302	4602
	Vd-X-12 h vs CK	199	102	301	
	Vd-X-24 h vs CK	814	1104	1918	
	Vd-X-48 h vs CK	948	1133	2081	
Vd-Z vs CK	Vd-Z-6 h vs CK	181	43	224	3896
	Vd-Z-12 h vs CK	193	279	472	
	Vd-Z-24 h vs CK	887	1128	2015	
	Vd-Z-48 h vs CK	820	1212	1185	
Vd-H vs CK	Vd-H-6 h vs CK	253	155	408	3227
	Vd-H-12 h vs CK	171	306	477	
	Vd-H-24 h vs CK	422	178	600	
	Vd-H-48 h vs CK	716	1026	1742	
Vd-W vs CK	Vd-W-6 h vs CK	61	114	175	2392
	Vd-W-12 h vs CK	134	189	479	
	Vd-W-24 h vs CK	301	178	626	
	Vd-W-48 h vs CK	367	745	1112	

**Fig. 4:**
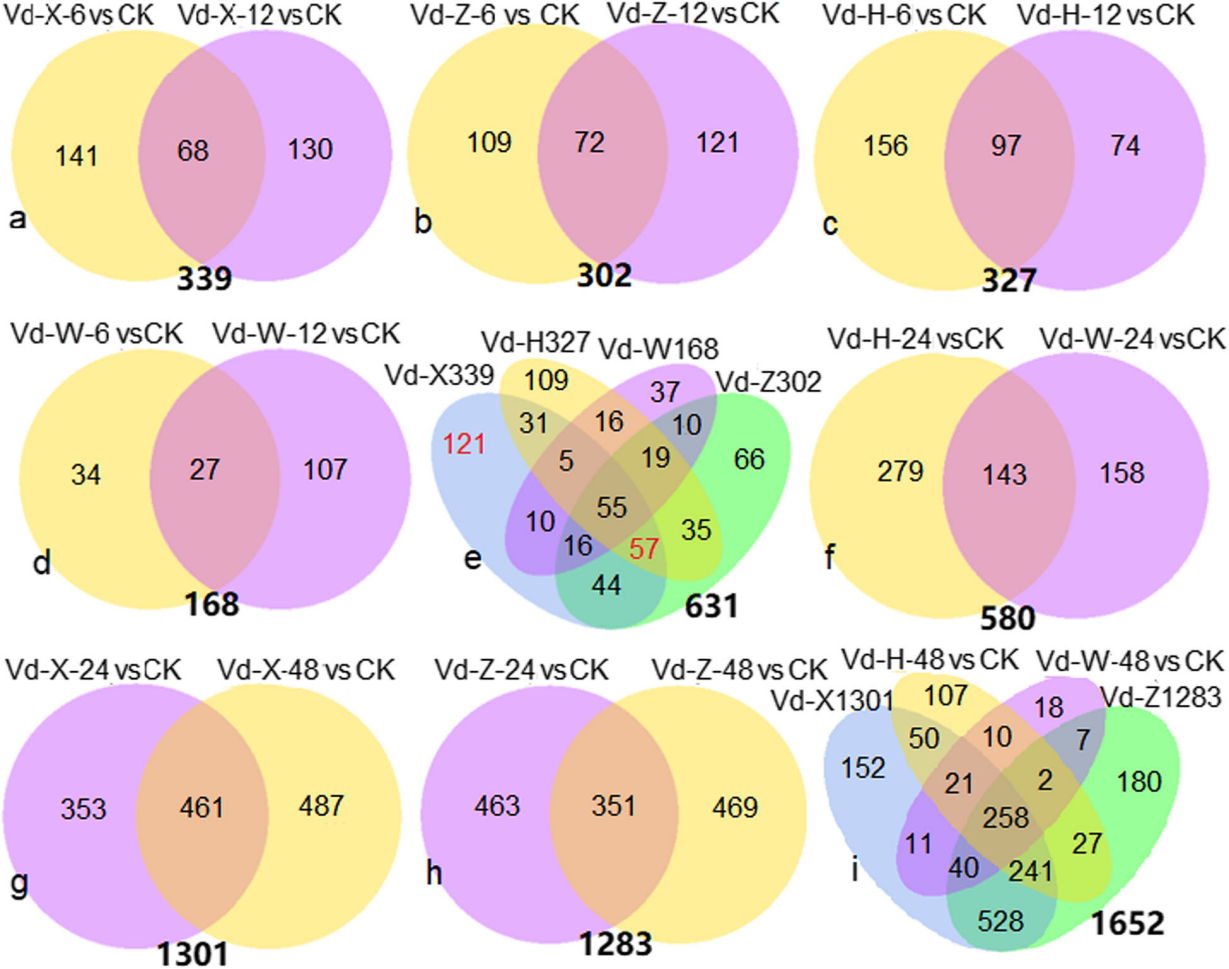
Overview of serial analysis of up-regulated DEGs identified in samples vs CK (Vd-0). **a**. Venn diagram of up-regulated DEGs in Vd-X-6 vs CK and Vd-X-12 vs CK. **b**. Venn diagram of up-regulated DEGs in Vd-Z-6 vs CK and Vd-Z-12 vs CK. **c**. Venn diagram of up-regulated DEGs in Vd-H-6 vs CK and Vd-H-12 vs CK. **d**. Venn diagram of up-regulated DEGs in Vd-W-6 vs CK and Vd-W-12 vs CK. **e**. Number of up-regulated DEGs identified in Vd-X vs CK (339), Vd-Z vs CK (302), Vd-H vs CK (327) and Vd-W vs CK (168). **f**. Venn diagram of up-regulated DEGs in Vd-H-24 vs CK and Vd-W-24 vs CK. **g**. Venn diagram of up-regulated DEGs in Vd-X-24 vs CK and Vd-X-48 vs CK. **h**. Venn diagram of up-regulated DEGs in Vd-Z-24 vs CK and Vd-Z-48 vs CK. **i**. Number of up-regulated DEGs identified in Vd-H-48 h (716), Vd-W-48 h vs CK (367), Vd-X vs CK (1201) and Vd-Z vs CK (1283). The Venn diagram in (**a**, **b**, **c**, **d**, **e**, **f**) represent serial analysis of up-regulated DEGs by comparing *V. dahliae* samples in the groupIwith CK. The Venn diagram in (**g**, **h**, **i**) represent serial analysis of up-regulated DEGs by comparing *V. dahliae* samples in the groupIIwith CK

To determine the genes of *V. dahliae* interacted with root exudates, the up-regulated genes in Vd-X, Vd-Z, Vd-H and Vd-W samples in the group I were examined, respectively. By combining up-regulated DEGs in Vd-6 vs CK and Vd-12 vs CK, a total of 339, 302, 327 and 168 DEGs were acquired in Vd-X vs CK, Vd-Z vs CK, Vd-H vs CK and Vd-W vs CK, respectively (Fig. 4a, b, c, d). These DEGs (339, 302, 327, 168) were combined together to get 631 DEGs (Fig. 4e). Although Vd-H-24 h and Vd-W-24 h were clustered in the groupI, they were analyzed separately because the number of up-regulated genes in Vd-H-24 h (422) and Vd-W-24 h (301) were obviously greater than other samples in group I (Table 3). By combining up-regulated DEGs in Vd-H-24 h vs CK (422) and Vd-W-24 h vs CK (301), a total of 580 DEGs were obtained (Fig. 4f).

The up-regulated genes in Vd-X, Vd-Z, Vd-H and Vd-W samples in the group II were also examined, respectively. By combining up-regulated DEGs in Vd-24 vs CK and Vd-48 vs CK, a total of 1301 and 1283 DEGs were acquired in Vd-X vs CK and Vd-Z vs CK (Fig. 4g, h), respectively. When these DEGs (1301, 1283) were combined together with DEGs in Vd-H-48 vs CK (716) and Vd-W-48 vs CK (367) comparisons, a total of 1652 DEGs were obtained (Fig. 4i).

### Gene ontology analyses of DEGs

**Fig. 5:**
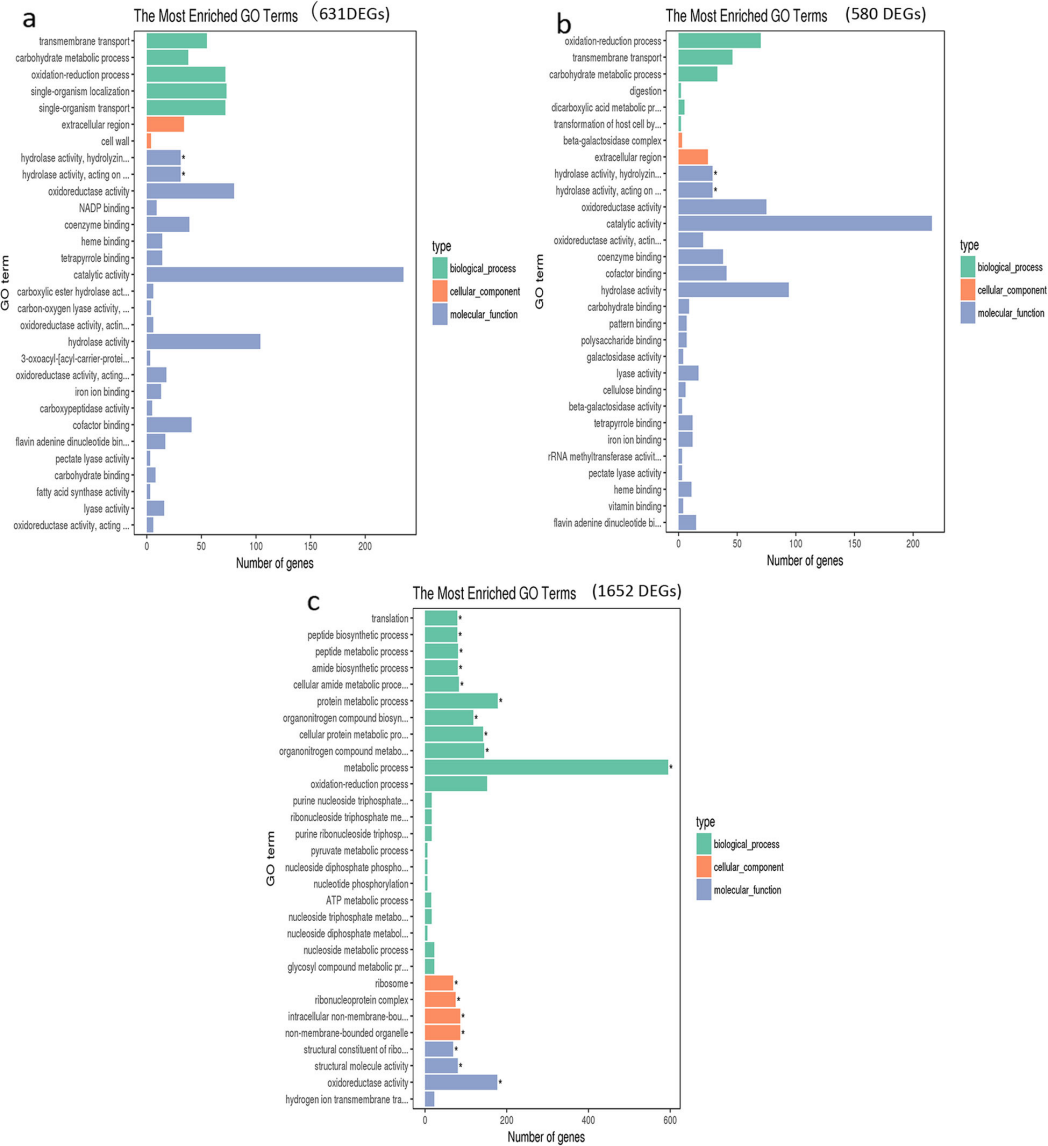
The most enriched GO terms of the up-regulated DEGs in *V. dahliae* samples vs CK. **a**. The most enriched GO terms of 631 up-regulated genes in samples of groupI (Vd-X, Vd-Z, Vd-H and Vd-W at 6 h and 12 h of cultured). **b**. The most enriched GO terms of 580 up-regulated genes in samples of groupI (Vd-H-24 h and Vd-W-24 h). **c**. The most enriched GO terms of 1652 up-regulated genes in samples of groupII (Vd-H-48 h, Vd-W-48 h, Vd-X and Vd-Z at 24 h and 48 h of cultured)

To further understand the function of these DEGs, we performed gene ontology (GO) analyses to classify the up-regulated genes in group I and group II samples, respectively. For the group I, up-regulated DEGs (631) were mainly enriched in molecular function category (Fig. 5a; Additional file 4: Table S2). ‘hydrolase activity, hydrolyzing O-glycosyl compounds’ (*p* = 1.22E-05), ‘hydrolase activity, acting on glycosyl bonds’ (*p* = 2.15E-05) and ‘oxidoreductase activity’ (*p* = 0.000309) were the top three significantly enriched terms in the molecular function category. ‘transmembrane transport’ (*p* = 3.77E-05), ‘carbohydrate metabolic process’ (*p* = 0.001034), ‘oxidation-reduction process’ (*p* = 0.001933) were the top three significantly enriched terms in the biological process category. ‘extracellular region’ (*p* = 0.000219) is the most significantly enriched term in the cellular component category. The enriched terms of 580 DEGs in Vd-H-24 vs CK combined with Vd-W-24 vs CK comparisons (Fig. 5b; Additional file 4: Table S2) were similar to that of 631 DEGs (Fig. 5a), suggesting that Vd-H-24 and Vd-W-24 were at the same stage of *V. dahliae* development as the other samples in group I. Therefore, it can be inferred that the response of *V. dahliae* to island cotton was more prolonged compared with upland cotton.

For DEGs (1652) that were up-regulated in the group II, the GO terms changed greatly compared with the group I (Fig. 5c; Additional file 4: Table S2). These DEGs were mainly enriched in biological process category. ‘translation’ (*p* = 1.67E-10), ‘peptide biosynthetic process’ (*p* = 4.18E-10) and ‘peptide metabolic process’ (*p* = 6.47E-10) were the top three significantly enriched terms in the biological process category. ‘structural constituent of ribosome’ (*p* = 3.70E-12) was the most significantly enriched term in molecular function category. ‘ribosome’ (*p* = 6.66E-12) and ‘ribonucleoprotein complex’ (*p* = 1.81E-06) were the significantly terms enriched in the component category.

It was notable that some genes were related to hydrolase activity, hydrolyzing O-glycosyl compounds and transmembrane transport which have been reported to be closely related to the pathogenicity of fungi, such as cell wall-degrading enzymes, sugar transporter and MFS transporter [52–55]. This GO terms were significantly enriched in samples of group I, suggesting that these samples were at the critical stage of *V. dahliae*-cotton interaction (6 h and 12 h). Therefore, *V. dahliae* samples at 6 h and 12 h were used for further analysis.

**Fig. 6:**
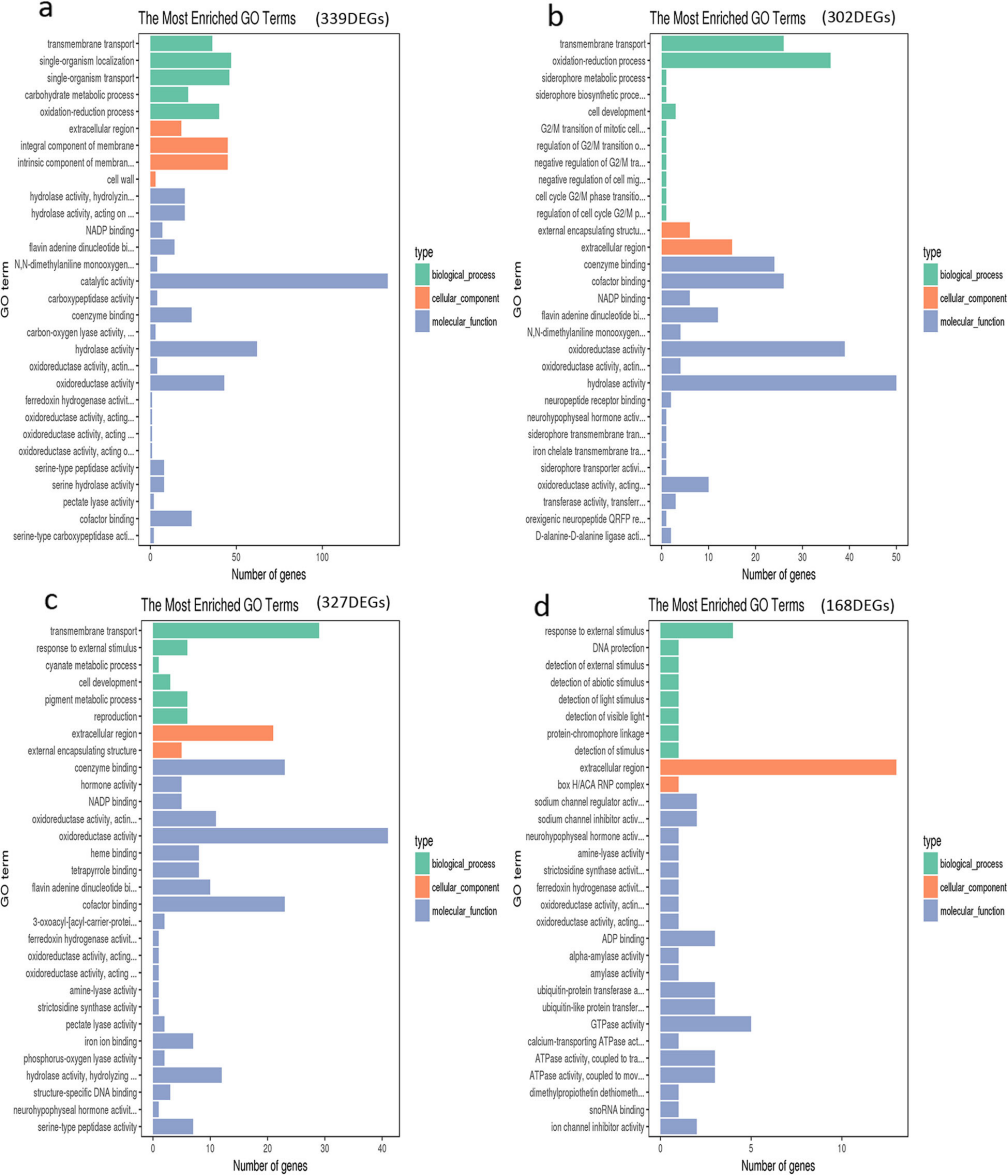
The most enriched GO terms of the up-regulated genes at 6 h and 12 h. **a**. The enriched GO terms of up-regulated genes in Vd-X vs CK (339). **b**. The enriched GO terms of up-regulated genes in Vd-Z vs CK (302). **c**. The enriched GO terms of up-regulated genes in Vd-H vs CK (327). **d**. The enriched GO terms of up-regulated genes in Vd-W vs CK (168)

In order to find the differences of *V. dahliae* interacted with different root exudates, we further performed the GO analyses to classify the up-regulated genes in Vd-X vs CK (339), Vd-Z vs CK (302), Vd-H vs CK (327), Vd-W vs CK (168), respectively (Fig. 6). In addition to Vd-W vs CK (Fig. 6; Additional file 5: Table S3), it was found that ‘transmembrane transport’ was the most significantly enriched term in all the other comparisons examined (Fig. 6a, b, c). Additionally, the enriched GO terms ‘coenzyme binding’, ‘NADP binding’, ‘cofactor binding’, ‘oxidoreductase activity’, ‘flavin adenine dinucleotide binding’, ‘extracellular region’ were commonly found in Vd-X (339), Vd-Z vs CK (302) and Vd-H vs CK (327) comparisons. However, ‘hydrolase activity, hydrolyzing O-glycosyl compounds’ was the most significantly enriched term in Vd-X vs CK (339) (Fig. 6a), but was not obviously enriched in Vd-Z vs CK (302), Vd-H vs CK (327) and Vd-W vs CK (168) (Fig. 6b, c, d).

**Fig. 7:**
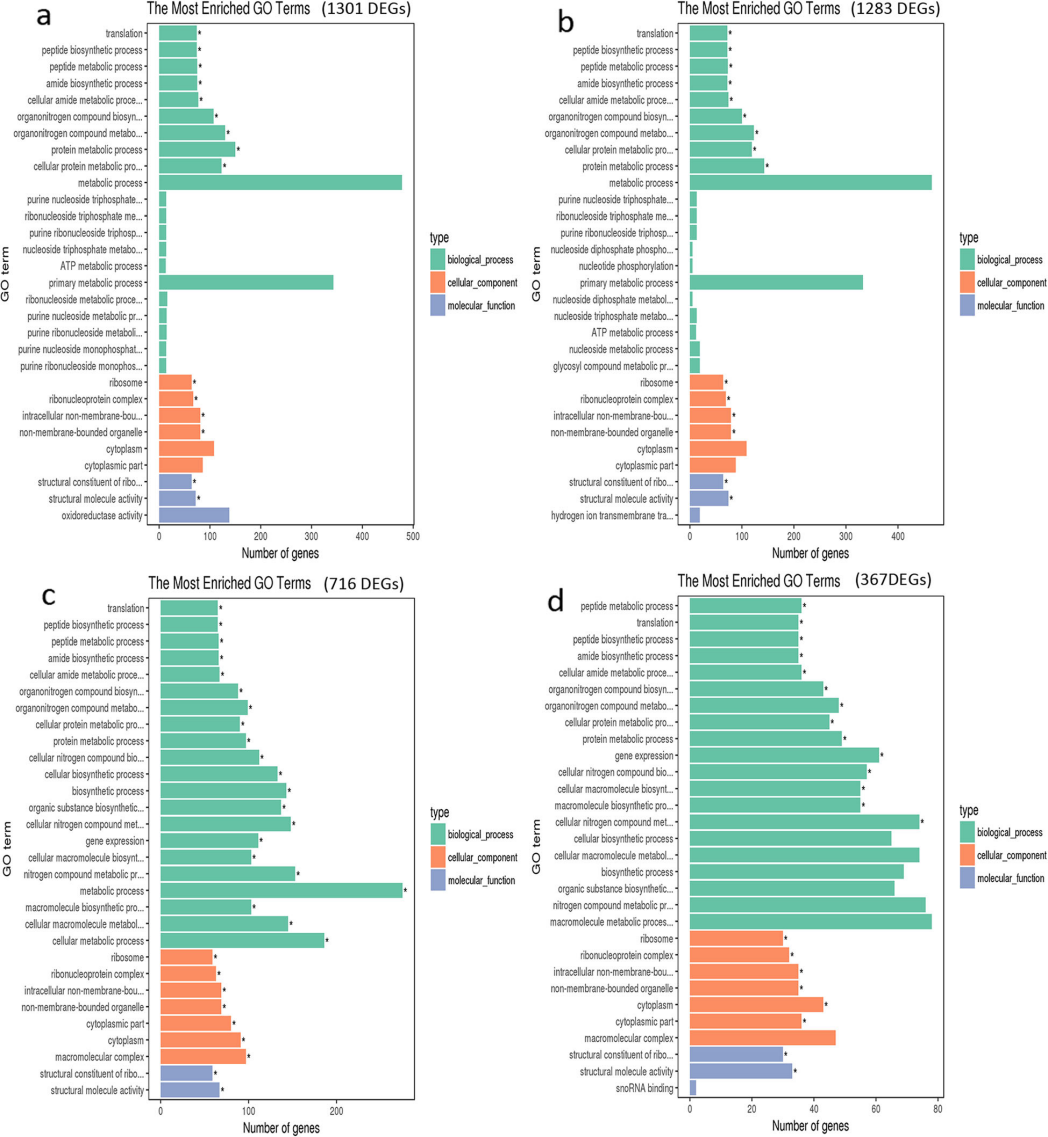
The most enriched GO terms of the up-regulated genes in group II, respectively. **a**. The enriched GO terms of up-regulated genes in Vd-X vs CK (1301). **b**. The enriched GO terms of up-regulated genes in Vd-Z vs CK (1283). **c**. The enriched GO terms of up-regulated genes in Vd-H-48 vs CK (716). **d**. The enriched GO terms of up-regulated genes in Vd-W-48 vs CK (367)

We also performed GO analyses to classify the up-regulated genes in Vd-X vs CK (1301), Vd-Z vs CK (1283), Vd-H vs CK (716), Vd-W vs CK (367), respectively (Fig. 7; Additional file 6: Table S4). As expected, the GO enriched terms of the up-regulated genes in Vd-X vs CK (1301), Vd-Z vs CK (1283), Vd-H-48 vs CK (716), Vd-W-48 vs CK (367) were very similar. It was found that ‘translation’, ‘peptide biosynthetic process’ and ‘peptide metabolic process’ were the top three significantly enriched terms in the biological process category. ‘ribosome’, ‘ribonucleoprotein complex’ and ‘intracellular non-membrane-bou’ were the top three significantly enriched term in the component category. ‘structural constituent of ribosome’ and ‘structural molecule activity’ were the significantly terms enriched in molecular function category. No ‘transmembrane transport’ and ‘hydrolase activity, hydrolyzing O-glycosyl compounds’ Go enriched terms were found in these samples of group II, again suggesting that 6 h and 12 h were the critical stage of *V. dahliae*-cotton interaction, while 24 h and 48 h were not.

### Genes response to root exudates from different cotton cultivars in *V.dahliae*

**Fig. 8:**
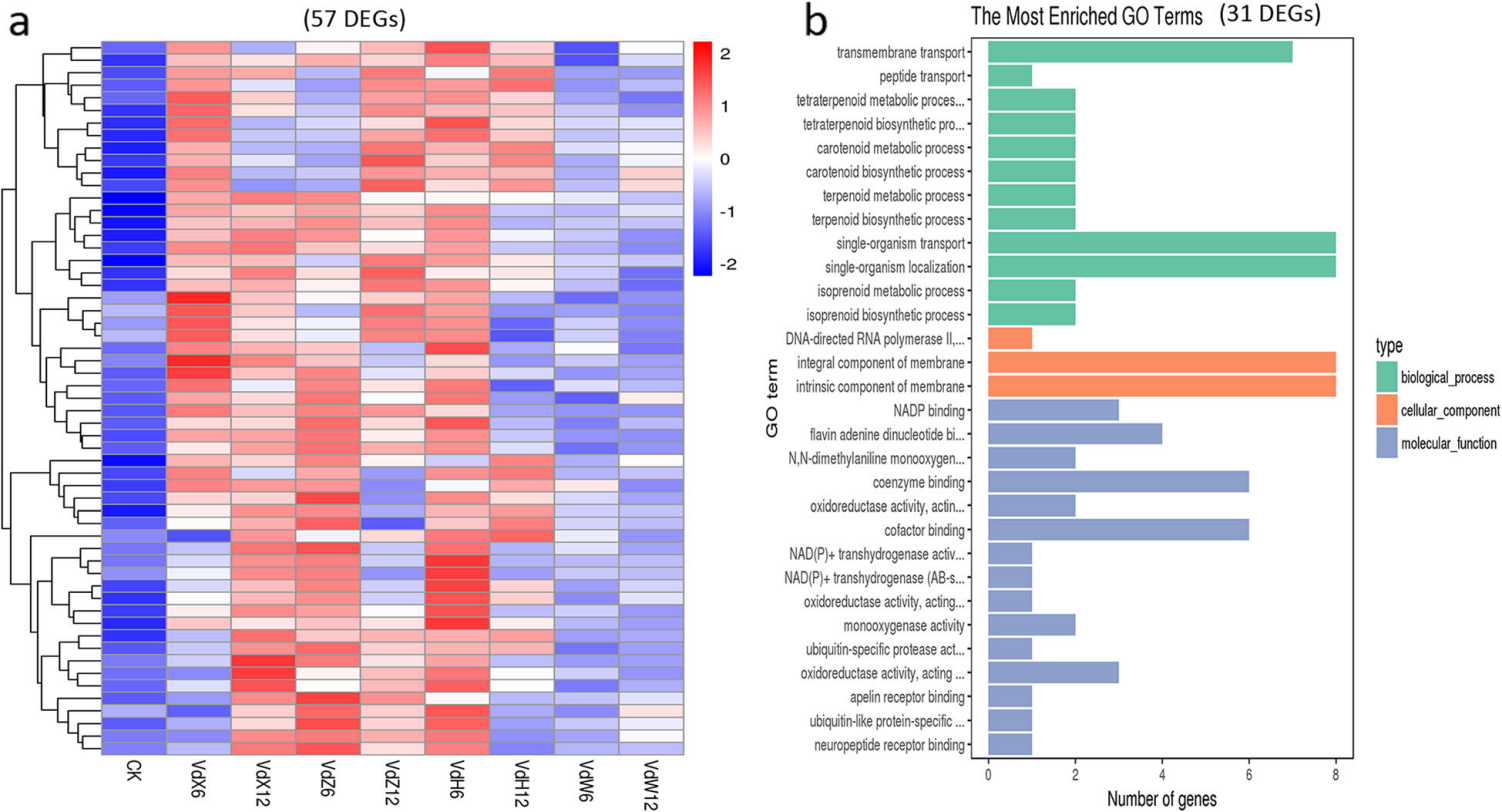
Heatmap and GO analyses of up-regulated genes in Vd-X, Vd-Z and Vd-H, respectively. **a**. Heatmap of 57 genes found to be up-regulated in Vd-X, Vd-Z and Vd-H at one or two time points of cultured (6 h and 12 h). The log-transformed expression values range from − 2 to 2. Red and blue bands represent high and low gene expression levels, respectively. **b**. The most enriched GO terms of the 31 DEGs with known functions

GO analyses for the up-regulated DEGs found that transmembrane transport was the most significantly enriched GO term in Vd-X vs CK (339), Vd-Z vs CK (302), Vd-H vs CK (327) comparisons, but not enriched in Vd-W vs CK (168), suggesting that genes related to this term were closely related to the initial steps of the roots infections. Several other GO enriched terms, ‘coenzyme binding’, ‘NADP binding’, ‘cofactor binding’, ‘oxidoreductase activity’, ‘flavin adenine dinucleotide binding’, ‘extracellular region’ were commonly enriched in Vd-X vs CK (339), Vd-Z vs CK (302), Vd-H vs CK (327), suggesting that genes related to these GO terms were also required for the initial steps of the roots infections. Although the main enriched GO terms were similar, the DEGs were quite different in Vd-X vs CK (339), Vd-Z vs CK (302) and Vd-H vs CK (327). Only 57 genes (Fig. 4e) were found to be commonly up-regulated in Vd-X, Vd-Z and Vd-H at the early stages of interaction. The Heatmap of 57 genes indicated that the expression level of these genes were obviously up-regulated in Vd-X, Vd-Z, and Vd-H at one or two time points of cultured, but not obviously up-regulated in Vd-W (Fig. 8a). These genes were considered as potential candidates for involvement in the initial steps of the roots infections. The 57 genes included 31 genes with known functions (Table 4), and 26 genes with unknown functions. Of 31 genes with known functions, it is notable that 7 genes were related to transmembrane transport (Fig. 8b; Additional file 7: Table S5), including 4 sugar transporter genes (VDAG_09835, VDAG_02051, VDAG_03649, VDAG_09983), 1 pantothenate transporter liz1 gene (VDAG_02269), 1 DUF895 domain membrane protein gene (VDAG_07864) and 1 Inner membrane transport protein yfaV gene (VDAG_00832) (Table 4). Few genes have been reported to be related to pathogenicity of *V. dahliae*, such as a gene encoding cyclopentanone 1,2-monooxygenase [18], two genes encoding thiamine transporter protein [56, 57]. Functional analysis for these candidate genes may be useful for the study of the molecular basis of *V. dahliae* interacted with cotton.

**Table 4:** Up-regulated genes with known functions in Vd-X, Vd-Z and Vd-H at early stages of interaction

Code	Gene ID	Enzyme name	FPKM value								
			CK	VdX6	VdX12	VdZ6	VdZ12	VdH6	VdH12	VdW6	VdW12
1	VDAG_02051	High-affinity glucose transporter ght2	29.4802	22.04664	84.23955	47.6086	60.97154	93.52616	103.058	46.62293	30.56167
2	VDAG_03649	Sugar transporter	0.338716	1.026522	0.972863	0.571231	1.115773	0.729055	1.128715	0.509536	0.50572
3	VDAG_09835	Hexose transporter	1.465257	3.968813	3.936814	4.569835	3.108347	4.246236	2.397604	1.989069	1.935044
4	VDAG_09983	Sugar transporter	0.826351	1.330852	1.620789	1.96333	0.809208	1.517935	1.816533	1.172747	1.108737
5	VDAG_00832	Inner membrane transport protein yfaV	1.328122	2.69694	3.987047	4.988756	2.544548	6.30185	3.71284	2.102835	2.608609
6	VDAG_00833	Thiol-specific monooxygenase	2.812437	3.372149	6.229075	6.864658	4.642015	6.132306	2.821518	3.31251	3.406964
7	VDAG_02269	Pantothenate transporter liz1	0.574735	10.51644	3.593005	0.612767	8.552547	5.701658	0.157546	0.281539	0.041829
8	VDAG_07864	DUF895 domain membrane protein	0	0.358107	0.365747	0.645067	0.105314	0.505642	0.342338	0.213305	0.133121
9	VDAG_01073	NAD (P) H-dependent D-xylose reductase	15.75744	20.66968	30.75152	33.81787	26.80472	28.37045	28.33363	16.91421	18.73819
10	VDAG_01137	Thiamine thiazole synthase	614.5958	1120.375	879.3086	769.972	1351.333	1043.097	1213.615	780.4955	914.2021
11	VDAG_01672	Conidial development protein fluffy	12.03245	22.95618	16.67069	14.22671	23.65364	22.70368	24.9867	13.75736	15.00116
12	VDAG_02162	Oviduct-specific glycoprotein	0.227984	0.693403	0.423062	0.620165	0.335718	0.664225	0.702286	0.371898	0.391474
13	VDAG_02175	Beta-glucosidase	0	0.230686	0.231927	0.374579	0.232406	0.403597	0.098718	0.036308	0.102984
14	VDAG_02633	Beta-lactamase family protein	3.050937	6.146087	5.612143	6.318416	5.777795	7.129429	6.223959	3.304537	4.690835
15	VDAG_02843	Fibronectin	2.255216	3.641777	4.31623	4.346089	2.988267	4.024384	4.227908	3.217397	3.142531
16	VDAG_02844	Ubiquitin carboxyl-terminal hydrolase	1.953236	4.647556	3.491474	2.867615	4.295582	3.838786	3.769097	2.859783	2.493258
17	VDAG_03942	Beta-lactamase family protein	3.714124	111.1741	18.61932	8.608223	65.63955	58.8475	0.267788	5.385614	1.313926
18	VDAG_03943	Cyclopentanone 1,2-monooxygenase	3.091277	213.913	27.61544	13.96051	120.0825	92.52951	0.866178	8.16749	2.187358
19	VDAG_04707	Helicase SWR1	81.01633	152.1582	107.6387	110.2411	147.0122	139.7217	137.8717	105.301	132.1303
20	VDAG_05314	N-(5-amino-5-carboxypentanoyl)-L-cysteinyl-D-valine synthase	9.774005	25.65614	14.10339	15.6124	19.11988	27.47597	19.27068	15.58876	16.02938
21	VDAG_05458	Acetylxylan esterase	0.553047	2.897187	2.187976	2.776539	2.587627	1.928449	0.968931	0.862581	0.885039
22	VDAG_06953	Kinesin light chain	0.425595	1.336314	0.641706	0.95834	1.168227	1.627748	1.075756	0.38034	0.897279
23	VDAG_08600	Thiopurine S-methyltransferase family protein	28.38327	41.42097	71.57507	56.80394	59.66433	60.43575	62.81802	37.98759	38.88032
24	VDAG_08689	Retinol dehydrogenase	1.932819	2.608155	6.598116	8.790265	6.5672	4.225019	3.010194	3.177277	3.647746
25	VDAG_08954	Carboxylic ester hydrolase	0.956565	3.376228	5.336164	4.806672	3.062654	6.82588	2.173958	2.390473	1.772136
27	VDAG_08979	URE2 protein	4.080627	6.68038	7.958957	8.914888	7.624858	8.220244	5.710169	4.310528	4.728051
26	VDAG_09114	Galactose oxidase	0.076631	0.245438	0.555276	0.588735	0.221988	0.772877	0.178927	0.184511	0.193227
28	VDAG_09269	NAD (P) transhydrogenase	1.434656	5.410515	2.93751	2.364836	2.830261	3.377882	1.639596	1.075059	1.322266
29	VDAG_09707	Amidase	0.340497	0.597686	1.138296	0.675114	0.853334	1.090583	0.668288	0.390384	0.487727
30	VDAG_10195	Vacuolar protein sorting-associated protein	10.90674	26.16851	16.17757	16.16115	23.07354	25.98232	21.03424	16.03954	16.69196
31	VDAG_10402	Isoamyl alcohol oxidase	1.289472	3.463787	3.225775	3.469081	3.14358	3.691591	2.373917	2.260947	2.547368

### Genes response to root exudates from susceptible cotton cultivar in *V. dahliae*

GO analyses for the up-regulated DEGs found that ‘hydrolase activity, hydrolyzing O-glycosyl compounds’ was the most significantly enriched term in molecular function category in Vd-X (339) (*p* = 8.78E-05) (Fig. 6a), but not in Vd-Z (302), Vd-H (327), Vd-W (168) (Fig. 6b, c, d) suggesting that genes related to this term would be contribute to the pathogenesis of *V. dahliae*. A total of 20 genes related to this term were found in Vd-X (339), including 16 genes (1–16) reported to be related to cell wall degradation (Table 5) [58].

**Table 5:** List of 20 genes in ‘hydrolase activity, hydrolyzing O-glycosyl compounds’ term

Code	Gene ID	Enzyme name	FPKM value				
			CK	VdX6	VdX12	VdX24	VdX48
1	VDAG_01555	Alpha-glucosidase	0.525139	1.351249	0.631004	0.348396	0.365856
2	VDAG_01781	Polygalacturonase	4.42107	9.593065	6.264324	4.446467	3.902134
3	VDAG_01866	Xylosidase/arabinosidase	2.999633	6.737425	3.158829	1.499116	1.280038
4	VDAG_02175	Beta-glucosidase	0	0.230686	0.231927	0.113611	0.277088
5	VDAG_02469	Glucan 1,3-beta-glucosidase	9.092852	19.9074	14.55665	10.28952	7.637805
6	VDAG_02542	Beta-glucosidase	1.740641	3.559025	2.760439	1.618581	1.613549
7	VDAG_03038	Trehalase	4.037018	10.34559	4.171279	3.42203	2.780894
8	VDAG_03553	Alpha-N-arabinofuranosidase	2.017713	2.840681	4.043869	1.883232	2.186114
9	VDAG_03526	Alpha-glucuronidase	3.077552	7.649031	3.025971	2.462925	2.180948
10	VDAG_03790	Endo-1,4-beta-xylanase	0.988496	3.303921	1.049834	1.031276	1.380458
11	VDAG_05708	Endoglucanase II	0.335166	0.833922	1.422978	0.420705	1.30643
12	VDAG_06072	alpha-1,2-Mannosidase	9.973456	12.06809	20.52097	6.852838	6.954053
13	VDAG_06165	Endo-1,4-beta-xylanase	0.808652	1.606539	1.930602	1.34546	0.837317
14	VDAG_07983	Mixed-linked glucanase	2.264495	5.11995	2.258442	0.964882	0.623151
15	VDAG_09516	Glucanase	0.565726	1.419057	0.616539	0.988646	0.415697
16	VDAG_09739	Galactan 1,3-beta-galactosidase	0	0.361516	0.129547	0.044338	0.086698
17	VDAG_02162	Oviduct-specific glycoprotein	0.227984	0.693403	0.423062	0.287278	0.208611
18	VDAG_05270	Ankyrin repeat and protein kinase domain-containing protein	0.050289	0.467369	0.237746	0.317916	0.584707
19	VDAG_07990	Secreted protein	0.318378	0.605114	1.132354	0.273572	0.134914
20	VDAG_08742	RTA1 protein	1.754573	3.368212	2.766573	3.690469	2.091908

**Fig. 9:**
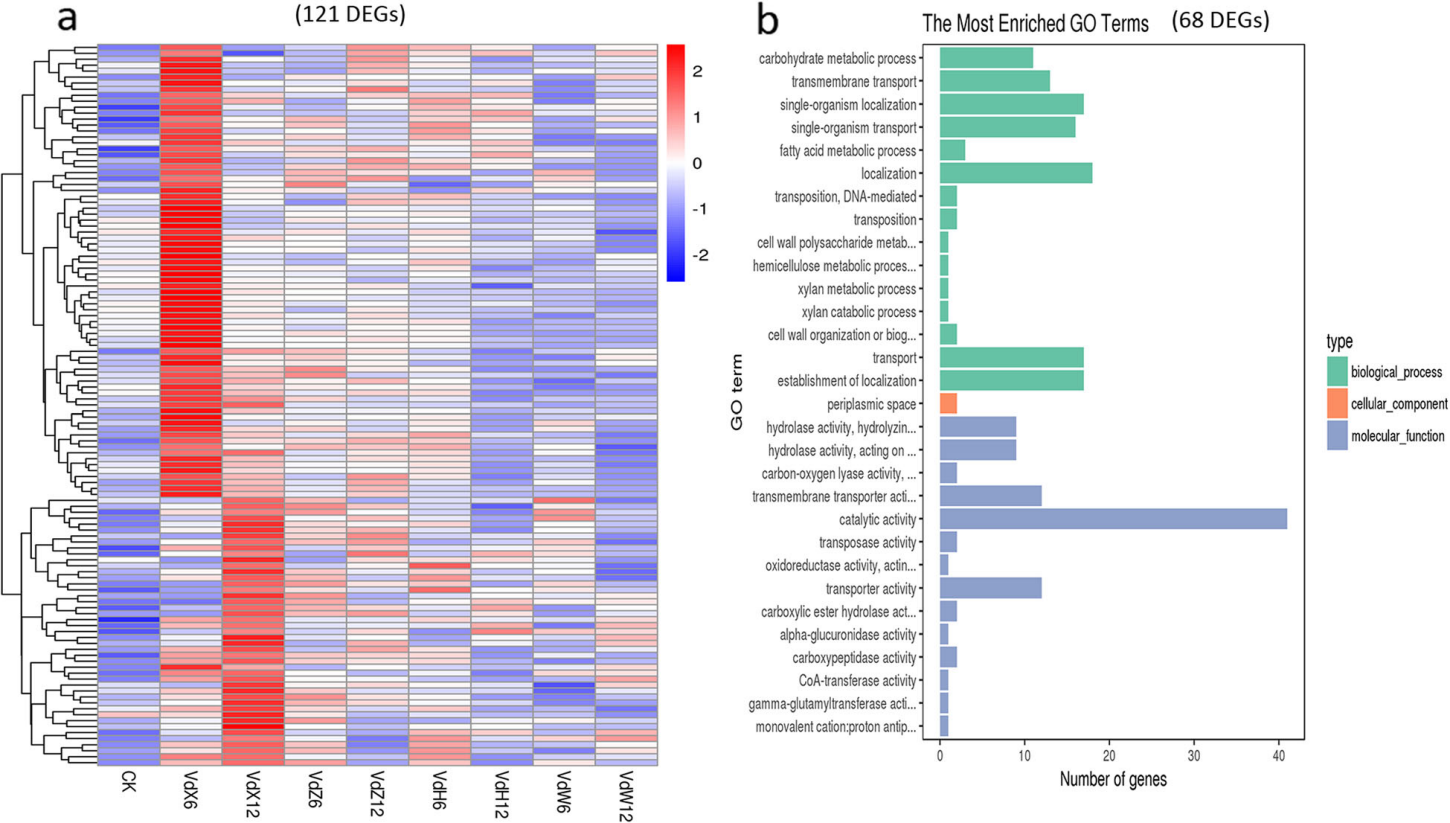
Heatmap and GO analyses of up-regulated genes only in Vd-X at 6 h or 12 h. **a**. Heatmap of 121 genes found to be up-regulated only in Vd-X at 6 h or 12 h of cultured. The log-transformed expression values range from − 2 to 2. Red and blue bands represent high and low gene expression levels, respectively. **b**. The most enriched GO terms of the 68 DEGs with known functions

A total of 121 DEGs unique to Vd-X (Fig. 4e) whose expression were up-regulated only in root exudates from susceptible cotton cultivar (X) were thought to be the candidate genes related to pathogenesis of *V. dahliae*. The Heatmap of 121 genes indicated that the expression level of these genes were obviously up-regulated in Vd-X, and only few genes were also up-regulated in Vd-Z, Vd-H, and Vd-W at one or two time points of cultured (Fig. 9a). The 121 DEGs included 68 genes with known functions (Table 6), 57 genes with unknown functions. Of 68 DEGs with known functions, it is notable that 9 genes related to hydrolase activity, hydrolyzing O-glycosyl compounds (Fig. 9b; Additional file 8: Table S6) encode cell wall-degrading proteins, including endo-1,4-beta-xylanase (VDAG_03790, VDAG_06165), xylosidase/arabinosidase (VDAG_01866), mixed-linked glucanase (VDAG_07983), glucanase (VDAG_09516), trehalase (VDAG_03038), Alpha-glucosidase (VDAG_01555), Alpha-glucuronidase (VDAG_03526), Alpha-N-arabinofuranosidase (VDAG_03553), 13 genes were related to transmembrane transport, including 6 sugar transporter genes (VDAG_07141, VDAG_04513, VDAG_08286, VDAG_09121, VDAG_07563, VDAG_03714), 3 vitamin transporter genes (VDAG_01193, VDAG_09734, VDAG_08086), 2 oligopeptide transporter (VDAG_06060, VDAG_05125), 1 MFS transporter gene (VDAG_09088), 1 quinate permease gene (VDAG_02089). Functional analysis for these candidate genes may be useful for the study of the pathogenicity molecular basis of *V. dahliae*.

**Table 6:** Up-regulated Genes with known functions only in Vd-X at early stages of interaction

Code	Gene ID	Enzyme name	FPKM value								
			CK	VdX6	VdX12	VdZ6	VdZ12	VdH6	VdH12	VdW6	VdW12
1	VDAG_01555	Alpha-glucosidase	0.525139	1.351249	0.631004	0.605564	0.620046	0.590879	0.45985	0.402997	0.450216
2	VDAG_01866	Xylosidase/arabinosidase	2.999633	6.737425	3.158829	3.239391	2.54392	3.426193	2.85372	2.522221	2.049945
3	VDAG_03038	Trehalase	4.037018	10.34559	4.171279	3.328726	3.723864	3.169099	3.525587	2.688863	2.205004
4	VDAG_03553	Alpha-N-arabinofuranosidase	2.017713	2.840681	4.043869	3.204145	2.274602	3.487533	2.467085	2.319605	1.751206
5	VDAG_03526	Alpha-glucuronidase	3.077552	7.649031	3.025971	2.906985	3.357615	3.521792	2.073764	2.32711	2.3016
6	VDAG_03790	Endo-1,4-beta-xylanase	0.988496	3.303921	1.049834	0.937063	0.564055	0.872658	0.73547	0.559991	0.670659
7	VDAG_06165	Endo-1,4-beta-xylanase	0.808652	1.606539	1.930602	1.213314	1.249954	1.229588	1.647082	0.870066	1.601574
8	VDAG_07983	Mixed-linked glucanase	2.264495	5.11995	2.258442	2.232694	2.349181	1.691225	0.893242	1.840287	1.631545
9	VDAG_09516	Glucanase	0.565726	1.419057	0.616539	0.766083	1.168563	0.644183	0.590824	0.426934	0.643502
10	VDAG_01193	High-affinity nicotinic acid transporter	0.922962	4.474884	2.389495	1.367505	2.293024	1.158877	1.052356	1.003427	0.855293
11	VDAG_02089	Quinate permease	0.246652	1.239759	0.27095	0.039896	0.305545	0.133467	0.052949	0.090075	0
12	VDAG_02826	Voltage-gated potassium channel subunit beta-1	0.52636	1.770148	1.55752	0.539201	0.500666	0.471194	0.446343	0.28894	0.387249
13	VDAG_03714	Sugar transporter	0	0.329793	0.192273	0.038948	0.105726	0.067001	0.051691	0.087935	0.148554
14	VDAG_04513	Hexose transporter protein	2.194243	6.031784	2.31866	2.734094	2.48747	2.407533	1.509354	1.42593	1.515528
15	VDAG_05125	Oligopeptide transporter 1	0.10654	0.48534	0.340637	0.244306	0.11979	0.146367	0.035656	0.11036	0.100641
16	VDAG_06060	Oligopeptide transporter 2	1.273316	3.507472	1.424591	1.091823	1.400967	1.407274	0.863739	0.954953	0.987525
17	VDAG_07141	H+/hexose cotransporter 1	1.174547	3.193565	2.110352	1.578545	2.020917	2.37435	1.543719	1.33677	1.028067
18	VDAG_07563	Sugar transporter STL1	3.617449	23.46294	4.627167	4.448156	3.630124	4.030519	3.522212	2.785485	1.485532
19	VDAG_08086	Vitamin H transporter 1	1.611487	2.873923	3.84332	1.865509	3.010686	2.468418	1.583815	1.710399	2.150159
20	VDAG_09088	MFS transporter	0.338652	3.60446	0.393707	0.739129	0.402541	0.835313	0.099841	1.023219	0.125486
21	VDAG_09121	Maltose permease MAL31	2.060967	3.647271	3.112186	2.227495	2.585063	3.214763	2.716028	2.03735	2.722908
22	VDAG_09734	Major myo-inositol transporter iolT	8.604817	27.04882	11.02109	8.86173	9.040651	10.25533	5.441678	7.247084	5.418201
23	VDAG_00798	Calphotin	2.4298	4.795505	3.519256	3.892562	3.226339	4.260004	3.460753	2.902065	2.982781
24	VDAG_01176	4-coumarate-CoA ligase	0.286	1.403583	0.44009	0.381114	0.556027	0.320558	0.53875	0.105766	0.323503
25	VDAG_01341	Methylitaconate delta2-delta3-isomerase	0.899946	1.132172	2.138507	1.664966	1.364732	2.236922	1.509204	1.611162	1.286028
26	VDAG_01782	Pectinesterase family protein	4.733987	9.575896	5.053127	6.412238	6.100081	6.127981	5.954252	4.959662	6.786958
27	VDAG_01783	Modification methylase Sau96I	0.081859	0.188908	0.307139	0.230715	0.074369	0.265676	0.115725	0.059275	0.191795
28	VDAG_01869	Taurine catabolism dioxygenase TauD	10.85428	19.91771	15.68955	17.54684	14.0093	16.81377	17.27372	13.04782	16.37882
29	VDAG_01837	Metallo-beta-lactamase superfamily protein	0.716978	2.102556	2.371603	1.81465	1.748865	1.727576	0.957063	1.388916	1.043824
30	VDAG_03354	Pectate lyase	0.259957	0.618515	1.255778	0.930004	0.591007	0.526287	0.19059	0.121278	0.308629
31	VDAG_03792	Beta-fructofuranosidase	1.417489	5.825462	1.214082	1.161383	1.005343	1.333573	0.668952	0.822075	0.921293
32	VDAG_03800	Phosphate transporter	1.066441	0.959226	2.010011	1.410633	1.683093	1.10869	1.149276	1.183568	0.876848
33	VDAG_03894	Lipase	0.079272	0.063231	0.623916	0.415827	0.091218	0.167024	0.219462	0.179133	0.25405
34	VDAG_03891	Acetamidase	2.501319	6.074764	4.124551	3.080263	3.894677	2.879871	1.776109	1.402326	2.302258
35	VDAG_03941	Regulatory protein alcR	0.474947	2.525426	0.864933	1.051024	0.812903	0.673834	0.263023	0.168962	0.879413
36	VDAG_03970	4-trimethylaminobutyraldehyde dehydrogenase	1.11673	4.961036	1.515747	1.065759	1.558865	0.886226	0.667051	0.311335	0.77097
37	VDAG_04175	SAM and PH domain-containing protein	2.508312	4.896892	2.096234	2.206801	2.672685	2.29161	2.247836	2.221988	1.767456
38	VDAG_04685	AdhA	0	0.749311	0.363623	0.132737	0.418209	0.139558	0	0.184184	0
39	VDAG_04961	Aldehyde dehydrogenase	0.304878	0.985035	0.491127	0.246694	0.251239	0.381107	0.500614	0.036883	0.037001
40	VDAG_05050	Choline monooxygenase	1.342722	1.170244	3.332128	1.062762	0.474455	0.697089	0.450456	0.508567	0.251975
41	VDAG_05135	Carboxypeptidase S1	0	0.199664	0.217901	0.076268	0.038868	0.179948	0	0.046469	0.064716
42	VDAG_05297	3-alpha-(Or 20-beta)-hydroxysteroid dehydrogenase	0.502718	0.859157	1.524476	1.118018	1.276975	0.544056	0.668099	1.077308	0.380073
43	VDAG_05324	3-alpha-(Or 20-beta)-hydroxysteroid dehydrogenase	0.424632	2.728921	0.853371	0.702873	0.806426	0.900467	0.35943	0.584218	0.73203
44	VDAG_05455	Gamma-glutamyltranspeptidase	8.097854	14.62289	7.23067	6.837455	10.22524	8.804363	7.083837	7.027691	7.604183
45	VDAG_05780	Long-chain-alcohol oxidase	12.20917	23.38509	13.18572	13.34187	11.49875	13.68543	9.477272	10.87106	12.46451
46	VDAG_06126	Secreted protein	0.396382	2.540219	0.349184	0.221178	0.522292	0.419048	0.331605	0.146586	0.504571
47	VDAG_06334	Sodium/bile acid cotransporter 7-A	5.975553	10.44867	6.986222	7.134331	6.986681	5.925724	5.77749	6.149932	6.790046
48	VDAG_06756	Aldo-keto reductase yakc	0	0.064868	0.430866	0.068946	0	0.085674	0.06682	0	0
49	VDAG_06997	Epoxide hydrolase	0	0.343647	0.057084	0.180036	0.229805	0.229429	0.137809	0	0
50	VDAG_07057	Acetyl-coenzyme A synthetase	23.07698	71.91837	28.53978	25.50788	23.87777	25.41201	15.28279	11.00477	13.18577
51	VDAG_07158	ECM14 protein	1.312533	1.658648	2.765504	2.456603	2.03252	1.488009	0.944383	1.82507	1.283626
52	VDAG_07166	Carnitine O-palmitoyltransferase I	17.12393	31.32699	17.35379	13.93265	19.8167	20.48912	14.99808	12.56235	12.11036
53	VDAG_07544	Non-specific lipid-transfer protein	7.073779	14.02016	9.853166	11.52067	6.789217	7.23411	6.939248	6.207376	5.102574
54	VDAG_07681	ATP-binding cassette sub-family G member 5	0.280493	3.096587	0.36107	0.502172	0.490947	0.426678	0.394345	0.556072	0.142852
55	VDAG_07728	Adenine deaminase	0.871341	0.730222	1.862647	1.335189	0.613871	0.842805	0.83656	1.780561	0.44741
56	VDAG_07980	Peptide hydrolase	4.547497	11.54846	6.580591	4.869931	5.140242	5.641155	3.053364	4.299607	2.724793
57	VDAG_08067	Pectate lyase B	1.597656	3.549548	2.364415	2.864407	2.737817	2.960574	1.770214	2.357985	1.326954
58	VDAG_08286	Alpha-glucosides permease MPH2/3	3.761419	10.00247	2.846449	3.855032	2.85363	4.066861	2.265101	2.82029	0.99225
59	VDAG_08654	Acetyl-coenzyme A synthetase	6.311482	25.47375	11.4497	7.045619	9.681632	9.402415	4.885708	5.435872	4.019251
60	VDAG_08703	Alpha-1,2 mannosyltransferase KTR1	0.115893	0.332317	0.996546	0.726013	0.607646	0.444118	0.773915	0.233683	0.497122
61	VDAG_09082	Succinyl-CoA:3-ketoacid-coenzyme A transferase	4.461569	15.45278	8.700468	11.87125	6.812786	6.795712	2.9958	3.117604	3.772685
62	VDAG_09253	Sulfate transporter	0.621583	1.003273	1.925273	0.996173	0.614154	0.592044	0.590546	0.211895	0.651484
63	VDAG_09313	Alpha-ketoglutarate-dependent sulfonate dioxygenase	1.120543	1.596103	2.703301	1.520719	1.336662	2.082727	1.975342	1.398853	2.107069
64	VDAG_09583	Alcohol oxidase	0.0371	0.946964	0.029918	0.166105	0.170765	0.201409	0.030483	0.029096	0
65	VDAG_09712	Succinate/fumarate mitochondrial transporter	14.67152	66.4187	23.3401	11.31978	19.52469	18.52314	4.75121	5.791921	6.018953
66	VDAG_09813	C6 transcription factor RegA	0.355193	1.301947	0.457038	0.47428	0.543437	0.50541	0.322972	0.266267	0.23939
67	VDAG_10171	Fungal specific transcription factor domain-containing protein	1.890601	3.353588	3.570153	2.624426	2.242321	2.5453	1.963163	2.84132	2.766448
68	VDAG_10443	Rhamnogalacturonan lyase	2.396504	4.665757	2.848467	2.509498	2.189874	2.620633	1.958417	2.44632	1.750408

**Fig. 10:**
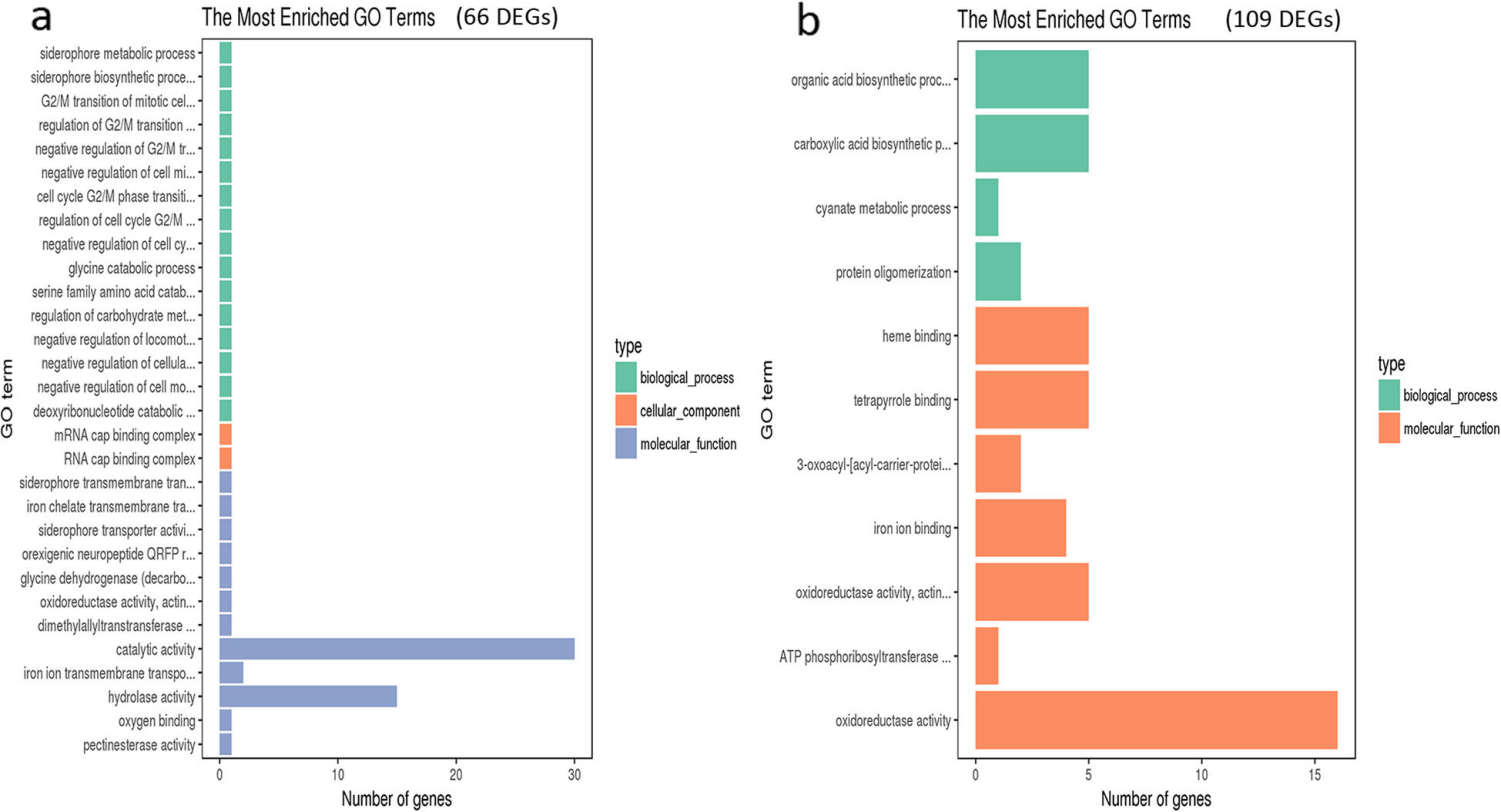
GO analyses of DEGs unique to Vd-Z vs CK and Vd-H vs CK. **a**. GO analysis of 66 DEGs unique to Vd-Z vs CK (307); **b**. GO analysis of 109 DEGs unique to Vd-H vs CK (327)

Additionally, GO analysis of 66 DEGs unique to Vd-Z (Fig. 10a) and 109 DEGs unique to Vd-H (Fig. 10b; Additional file 9: Table S7) did not find hydrolase activity, hydrolyzing O-glycosyl compounds and transmembrane transport enriched GO terms, suggesting that the number of DEGs related to hydrolase activity hydrolyzing O-glycosyl compounds and transmembrane transport in Vd-X vs CK (339) were higher than that in Vd-H vs CK (327) and Vd-Z vs CK (302) and these genes may be related to pathogenesis of *V. dahliae*.

### Genes related to development of *V. dahliae*

**Fig. 11:**
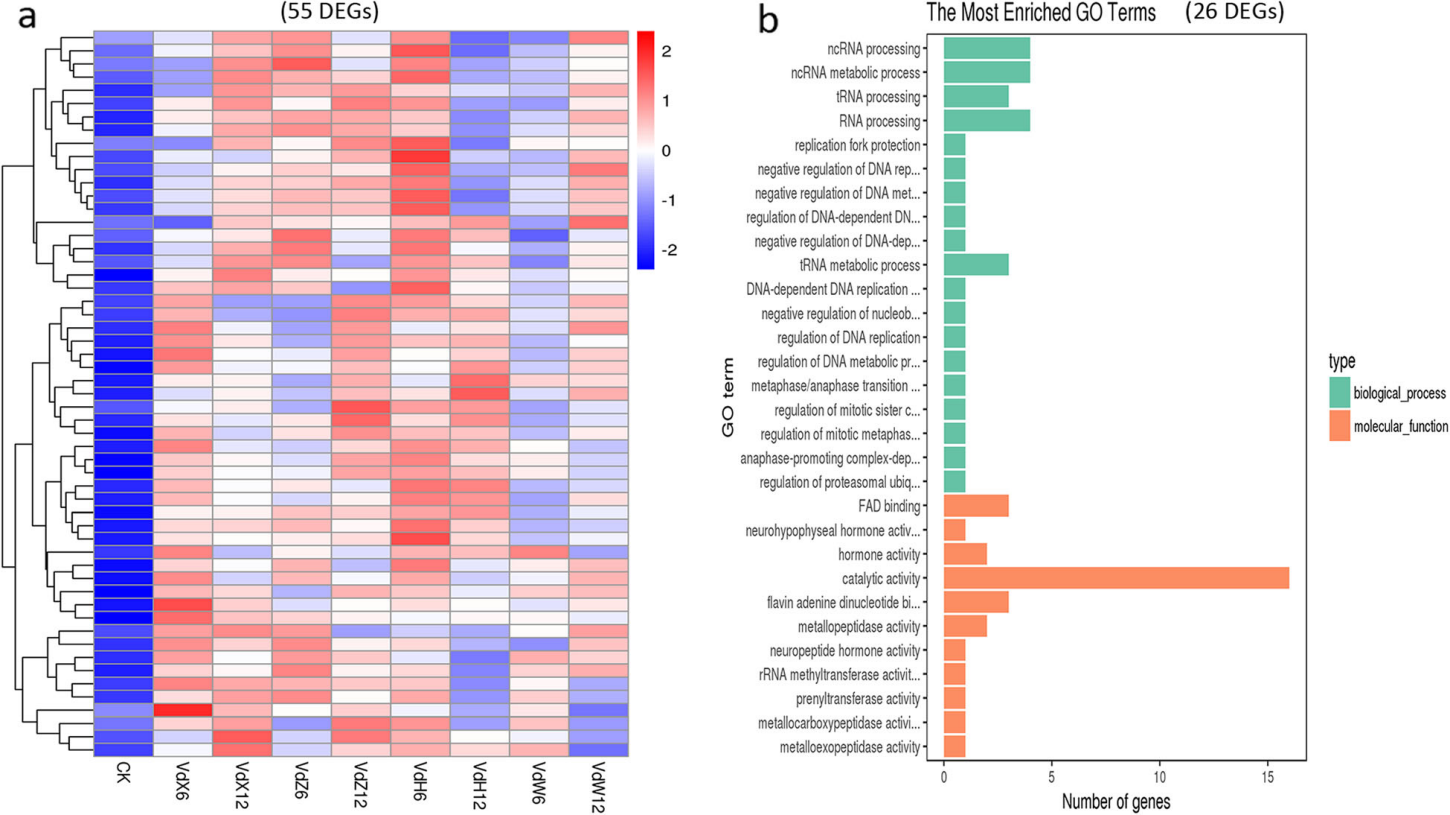
Heatmap and GO analyses of up-regulated genes in Vd-X, Vd-Z, Vd-H and Vd-W. **a**. Heatmap of 55 genes found to be up-regulated in Vd-X, Vd-Z, Vd-H and Vd-W at 6 h or 12 h of cultured. The log-transformed expression values range from − 2 to 2. Red and blue bands represent high and low gene expression levels, respectively. **b**. The most enriched GO terms of the 26 DEGs with known functions

A total of 55 genes (Fig. 4e) whose expression were up-regulated in Vd-X, Vd-Z, Vd-H and Vd-W were considered to be required for development of *V. dahliae*. The Heatmap of 55 genes indicated that the expression level of these genes were obviously up-regulated in Vd-X, Vd-Z, Vd-H and Vd-W at one or two time points of cultured (Fig. 11a), which was consistent with the Veen diagram results. The 55 genes included 26 genes with known functions and 29 genes with unknown functions. Of 26 DEGs with known functions (Table 7), it is notable that several genes were associated with FAD binding and RNA processing (Fig. 11b; Additional file 10: Table S8), such as VDAG_02063, VDAG_05832, VDAG_09806, VDAG_05829, VDAG_02981. Functional analysis for these candidate genes may be useful for the study of the molecular basis of *V. dahliae* development.

**Table 7:** Up-regulated genes with known functions in Vd-X, Vd-Z, Vd-H and Vd-W at 6 h or 12 h

Code	Gene ID	Enzyme name	FPKM value								
			CK	VdX6	VdX12	VdZ6	VdZ12	VdH6	VdH12	VdW6	VdW12
1	VDAG_01200	Multidrug resistance protein	2.574203	3.176818	8.456135	10.58305	4.440765	8.923448	3.200822	3.971342	5.1336
2	VDAG_02063	L-amino-acid oxidase	0.635877	0.5626	1.614603	1.483211	1.348462	1.682819	1.940567	0.826722	2.25874
3	VDAG_02178	Quinate permease	0.058306	0.627263	0.485426	0.606461	1.024123	0.628178	0.873477	0.321581	0.459276
4	VDAG_02520	Response regulator receiver domain-containing protein	11.43198	30.89575	40.29134	13.93595	49.39211	43.4858	14.60711	33.75135	13.91367
5	VDAG_02528	RNA-dependent RNA polymerase	2.550404	6.828783	5.918211	5.244344	7.547659	8.333781	6.355329	6.082573	5.05485
6	VDAG_02981	Methyltransferase domain-containing protein	1.048951	2.022277	2.645048	3.118421	2.771815	4.705878	1.726638	1.905085	4.283733
7	VDAG_03099	Glucan 1,3-beta-glucosidase	0.304888	1.159252	1.177232	1.289926	1.029788	1.617254	1.194427	0.743662	0.829034
8	VDAG_03536	YetA	14.37066	33.60962	24.66688	23.71565	30.52655	24.97965	27.41519	21.24693	27.49016
9	VDAG_03975	C6 zinc finger domain-containing protein	15.00863	29.21758	18.81392	18.84766	31.24542	29.8705	26.07178	21.38755	27.99133
10	VDAG_04598	Glycogenin-1	37.06267	68.97229	48.17265	45.46692	77.10089	69.11893	70.61616	54.23442	63.30747
11	VDAG_05008	Peptidase M20 domain-containing protein 2	0.951232	3.683467	2.85524	2.704868	2.485291	2.347381	2.428679	2.431284	2.273593
12	VDAG_05649	BNR/Asp-box repeat domain-containing protein	1.485317	5.698776	3.41501	3.134149	4.311877	5.442967	4.981001	3.789991	2.962473
13	VDAG_05829	Heat shock protein HSP98	2851.294	8213.736	5839.808	8385.88	7207.387	5492.246	3748.727	7855.11	6926.32
14	VDAG_05831	Phenylalanine ammonia-lyase	5.327448	10.4713	14.76473	18.7942	11.65773	24.15234	5.273917	8.005835	11.82046
15	VDAG_05832	FAD binding domain-containing protein	0.97946	5.375452	9.59148	13.22928	11.4021	29.036	1.738944	5.22337	11.88564
16	VDAG_05836	Para-hydroxybenzoate-polyprenyltransferase	0.16421	0.23375	2.238529	1.346959	3.046437	4.084031	0.142631	1.331249	1.254863
17	VDAG_06240	Phytanoyl-CoA dioxygenase	3.271209	4.96076	16.97013	14.02229	11.25868	20.8611	5.329389	5.988937	9.619971
18	VDAG_06907	E3 ubiquitin-protein ligase	19.52358	41.99442	38.90181	40.99749	42.84902	65.42277	43.04462	32.43004	37.12453
19	VDAG_07183	Carboxypeptidase A	0.590637	4.817832	2.275429	1.521062	2.023665	1.406215	0.779928	1.725569	0.451422
20	VDAG_07270	Mycocerosic acid synthase	0.563822	1.191726	1.288242	1.182283	0.764326	1.502613	1.056965	0.88995	0.998817
21	VDAG_07344	Cutinase	0	0.757969	0.60793	0.962218	1.396963	2.694802	0.561964	0.477185	1.374841
22	VDAG_07854	Maltose O-acetyltransferase	2.196192	3.946491	6.820049	7.200627	3.16318	6.916005	5.754764	2.764381	4.99843
23	VDAG_08529	Anaphase-promoting complex subunit 8	13.30899	23.23852	34.1324	40.32327	24.52103	40.96512	25.52018	20.15623	27.3556
24	VDAG_08712	Cyanide hydratase	0.511059	3.463236	24.39127	3.558516	18.23755	11.04813	5.794128	4.979065	1.917549
25	VDAG_09806	FAD binding domain-containing protein	0.850458	1.504143	1.687359	1.407464	1.919441	1.757326	2.404167	1.50623	1.979163
26	VDAG_10401	Integral membrane protein	1.080071	3.072609	4.37851	3.103026	2.956657	4.107363	3.177632	2.617038	2.934669

## Discussion

*V. dahliae* can survive for many years in soil and dead plant tissues, making Verticillium wilt difficult to control, which has been likened to a bottleneck in commercial crop productivity [53, 56]. Only limited studies have focused on pathogenicity-related molecular mechanisms in the fungus, In this study, RNA-Seq was firstly used to explore and compare the transcriptomic profiles of *V. dahliae* after cultured with root exudates from different cotton varieties. Statistical analysis of DEGs in *V. dahliae* samples vs CK (Vd-0) revealed that *V. dahliae* responded to all kinds of root exudates but was more responsive to susceptible cultivar than to tolerant and resistant cultivars. GO analysis revealed the enriched GO terms of up-regulated genes in Vd-X vs CK (339), Vd-Z vs CK (302), Vd-H vs CK (327) were similar. However, the up-regulated genes were quite different in these samples, and only 57 up-regulated genes were found to be common in Vd-X vs CK (339), Vd-Z vs CK (302) and Vd-H vs CK (327), suggesting that the molecular mechanism of the response of *V. dahliae* to different root exudates from three cotton cultivars was different. GO analysis also found that enriched GO terms of up-regulated genes in Vd-X (339) and Vd-Z (302) at 6 h and 12 h of cultured were obviously different from that of Vd-X (1031) and Vd-Z (1283) at 24 h and 48 h of cultured, suggesting that *V. dahliae* at 6 h and 12 h of cultured were at different growth stages compared with 24 h and 48 h of cultured. The discovery of enriched GO terms hydrolase activity, hydrolyzing O-glycosyl compounds and transmembrane transport in Vd-X vs CK (339) and Vd-Z vs CK (302) suggested that 6 h and 12 h were the critical stage of *V.dahliae-*cotton interaction for upland cotton. For Vd-H-24 h, the enriched GO terms were similar to that in Vd-H (327) at 6 h and 12 h of cultured, suggesting that the response of *V. dahliae* to island cotton was more prolonged compared with upland cotton. Additionally, the number of unique genes in *V. dahliae* cultured with root from susceptible cotton variety (121 DEGs) was much more than in *V. dahliae* cultured with tolerant (66 DEGs) and resistant varieties (109 DEGs), including more hydrolase activity hydrolyzing O-glycosyl compounds and transmembrane transport related DEGs, which can partly account for the reasons why *V. dahliae* can cause disease in susceptible cotton.

Plant pathogenic fungi can produce a range of cell wall-degrading enzymes to facilitate infection and colonization [59, 60], including cellulase, hemicellulase, pectinase, etc. Hydrolytic enzymes, particularly cellulases and pectinases, have been considered to be important for the expression of disease symptoms and pathogenesis of *V. dahliae* [61, 62]. The cell wall-degrading enzymes are virulence factors, such as such as xyloglucan-specific endoglucanase [63], fungal endopolygalacturonases [64], and also function as pathogen-associated molecular patterns (PAMPs). Specifically, the cell wall-degrading enzymes contain carbohydrate-binding modules (CBM), non-catalytic protein domains that are generally associated with carbohydrate hydrolases in fungi, which are known to act as elicitors of the PAMP-triggered immunity (PTI) response in oomycetes [65, 66]. In *V. dahliae*, two Glycoside hydrolase 12 (GH12) proteins, *VdEG1* and *VdEG3* acted as PAMPs to trigger cell death and PTI independent of their enzymatic activity in *Nicotiana benthamiana*.

Although cell wall-degrading enzymes have been received to be related to pathogenicity of fugus, but the direct molecular evidence was not sufficient. In this study, GO analyses for the up-regulated DEGs found that genes related to hydrolase activity, hydrolyzing O-glycosyl compounds was the most significantly enriched term in molecular function category for Vd-X (339), but not in Vd-Z (302), Vd-H (327), Vd-W (168), including 16 cell wall-degrading genes, suggesting these genes would be contribute to the pathogenesis of *V. dahliae*. Additionally, A total of 121 DEGs unique to Vd-X (339) whose expression were obviously up-regulated after cultured with root exudates from susceptible cotton cultivar, including 9 cell wall-degrading genes. These results provided a proof of the involvement of cell wall-degrading genes in the initial steps of the roots infections and likely in pathogenesis. Recently, functional studies of cell wall-degrading related genes by targeted gene knockout have been carried out to obtain mutants deficient in one or more these genes [60, 67], but were not conclusive due to the multigene families encoding these enzymes [68]. Therefore, it is important to detect which genes were responsible for the pathogenicity of *V. dahliae*. In this study, 16 cell wall-degrading related genes were significantly up-regulated in Vd-X at early stage of interaction, which can be used as the target genes for studying *V. dahliae* pathogenicity by gene knockout. Here some genes were up-regulated in *V. dahliae* cultured by water, maybe resulted from no nutrient in water. Perhaps the starvation of the fungus may induce expression of genes encoding cell wall-degrading enzymes [69].

The adaptation of *V. dahliae* inside the host plants requires a large number of channel proteins to control the absorption of nutrients across the plasma membrane [56]. Transport proteins are integral transmembrane protein that exist permanently within and span the membrane across which they transport substances. GO analyses found that transmembrane transport term was commonly enriched in Vd-X (339), Vd-Z (302), Vd-H (327), but not enriched in Vd-W (168) at 6 h and 12 h of cultured, suggesting that they were required for the initial steps of the roots infections. Seven genes related to transmembrane transport found to be up-regulated in *V. dahliae* cultured by different root exudates, and 13 genes related to this term were only up-regulated in *V. dahliae* cultured by root exudates from susceptible cultivar. The results exhibited that genes related to this term can respond quickly to cotton root exudates, especially to the susceptible cotton, suggesting that genes related to transmembrane transport may be associated with the initial steps of the roots infections and likely in pathogenesis. The content of carbohydrate and amount of amino acids in the root exudates of susceptible cultivar was distinctly more than resistant ones [42]. Thus, *V. dahliae* can obtain more nutrients to provide its growth in root exudates from susceptible cotton, which may be responsible for the higher expression of transmembrane transport genes at the early stage of interaction in *V. dahliae* cultured by root exudates from susceptible cotton. However, few transmembrane transport genes for nutrient acquisition have been identified from *V. dahliae*, and their involvement in the disease process is unknown.

In short, our study firstly revealed the transcriptomes of *V. dahliae* cultured with root exudates from different cotton cultivars. Our results provided the clear proof at the molecular level for the association of cell wall-degrading and transmembrane transport related genes with pathogenesis of *V. dahliae*. The results enriched the genomic information on *V. dahliae* in public databases, and laid a foundation for the evaluation and understanding the molecular mechanisms of *V. dahliae* interacted with cotton and pathogenicity. The paper provided a framework for further functional studies of candidate genes to develop better control strategies for the cotton wilt disease.

## Conclusions

In this study, we present the first comparative transcriptomic profiling analysis of *V. dahliae* responded to root exudates from a susceptible upland cotton cultivar, a tolerant upland cotton cultivar and a resistant island cotton cultivar. Our study provided a comprehensive examination of the biological processes in *V. dahliae* affected by different root exudates based on analysis of Gene Ontology (GO) terms of the differentially expressed genes, and described genes that were involved in the initial steps of the roots infections and likely in pathogenesis. Genes related to ‘hydrolase activity, hydrolyzing O-glycosyl compounds’ highly enriched in *V. dahliae* cultured by root exudates from susceptible cotton at early stage of interaction may be responsible for the pathogenicity of *V. dahliae*. Genes related to ‘transmembrane transport’ enriched in different root exudates, but not in water may be required for the initial steps of the roots infections. These expression data have advanced our understanding of key molecular events in the *V. dahliae* interacted with cotton, and provided a framework for further functional studies of candidate genes to develop better control strategies for the cotton wilt disease.

## Supplementary information

**Additional file 1: Figure S1.** Results of the Pearson’s correlation analysis of biological replicates.

**Additional file 2: Figure S2.** The expression profiles of 8 DEGs related to hydrolase activity hydrolyzing using their FPKM value.

**Additional file 3: Table S1.** Summary of RNA-seq reads mapped to the reference genome and uniquely mapped’s distribution.

**Additional file 4: Table S2.** The most enriched GO terms of the up-regulated DEGs in *V. dahliae* samples vs CK.

**Additional file 5: Table S3.** The most enriched GO terms of the up-regulated genes in Vd-X, Vd-Z, Vd-H and Vd-W at 6 h and 12 h of cultured, respectively.

**Additional file 6: Table S4.** The most enriched GO terms of the up-regulated genes in Vd-X, Vd-Z, Vd-H and Vd-W of group II, respectively.

**Additional file 7: Table S5.** The most enriched GO terms of the 31 DEGs with known functions.

**Additional file 8: Table S6.** The most enriched GO terms of the 68 DEGs with known functions.

**Additional file 9: Table S7.** GO analyses of DEGs unique to Vd-Z vs CK (307) and Vd-H vs CK (327).

**Additional file 10: Table S8.** The most enriched GO terms of the 26 DEGs with known functions.

## Data Availability

The gene sequences used for qRT-PCR analysis were available and download from the public database National Center for Biotechnology Information under the accession codes VDAG_10074, VDAG_01193, VDAG_01866, VDAG_03038, VDAG_03526, VDAG_04513, VDAG_07563, VDAG_08286 and VDAG_09088. All data supporting the findings of our study can be found within the manuscript and additional file tables. The transcriptomic data generated in current study are deposited in the NCBI SRA database with the BioProject accession:PRJNA545805.

## Abbreviations

CBM: Carbohydrate-Binding Modules
CT: Cycle Threshold
DEG: Differentially Expressed Gene
FPKM: Fragments Per Kilobase of exon per Million fragments mapped
GH: Glycoside Hydrolase
GO: Gene Ontology
H: Hai7124
MFS: the Major Facilitator Superfamily
PAMP: Pathogen-Associated Molecular Pattern
PTI: PAMP-Triggered Immunity
qRT-PCR: Quantitative Real-Time PCR
RNA-Seq: RNA Sequencing
*V. dahliae*: *Verticillium dahliae*
Vd: *Verticillium dahliae*
X: Xinluzao 8
Z: Zhongzhimian 2
