# Supplementary material for: Transcriptomic analysis of gene expression of Verticillium dahliae upon treatment of the cotton root exudates
Source: BMC Genomics. 2020 Feb 12;21:155. doi: 10.1186/s12864-020-6448-9 (PMC7017574; doi:10.1186/s12864-020-6448-9)
Supplement: Supplementary file 1 — Additional file 1: Figure S1. Results of the Pearson’s correlation analysis of biological replicates. [file 12864_2020_6448_MOESM1_ESM.docx]

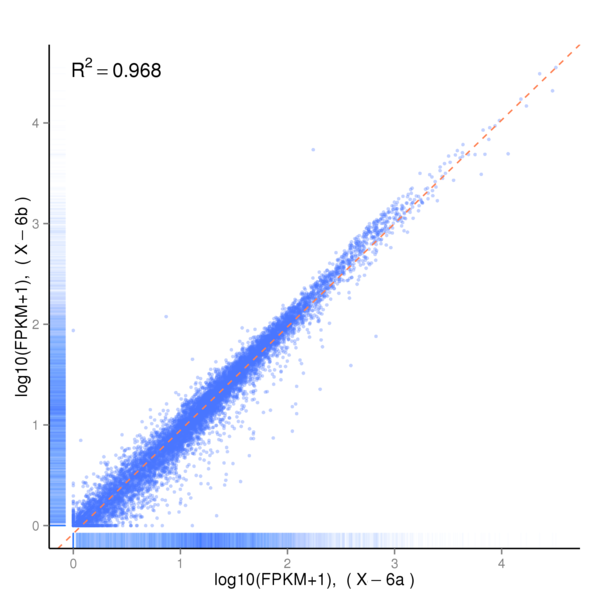

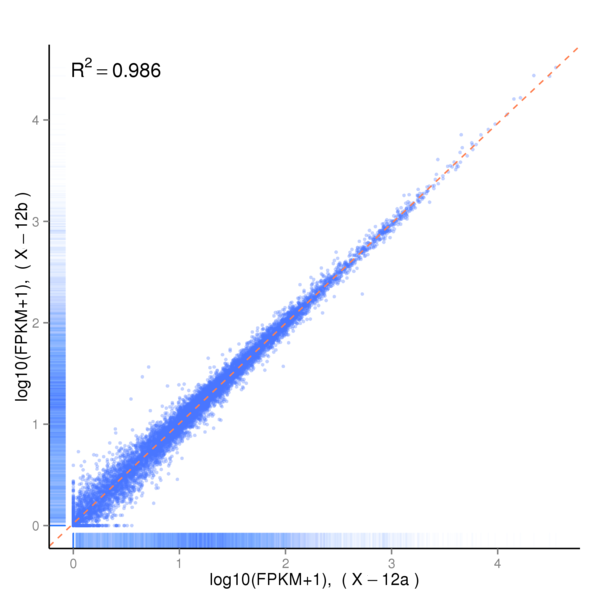


**Vd-X-12a vs Vd-X-12b**

**Vd-X-6a vs Vd-X-6b**


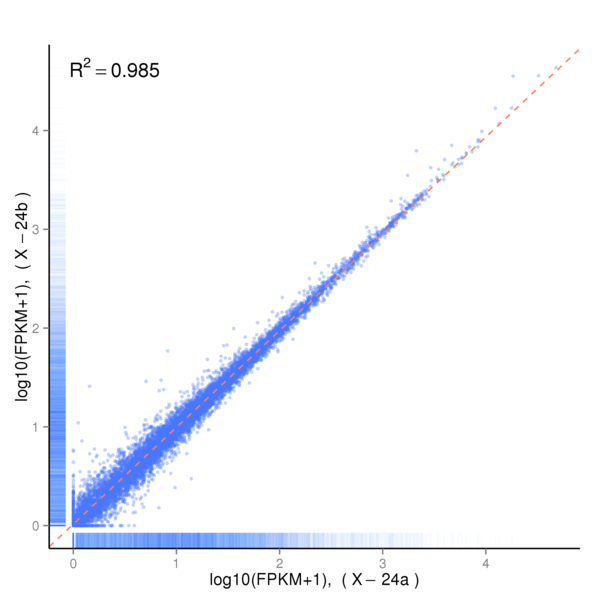


**Vd-X-24a vs Vd-X-24b**


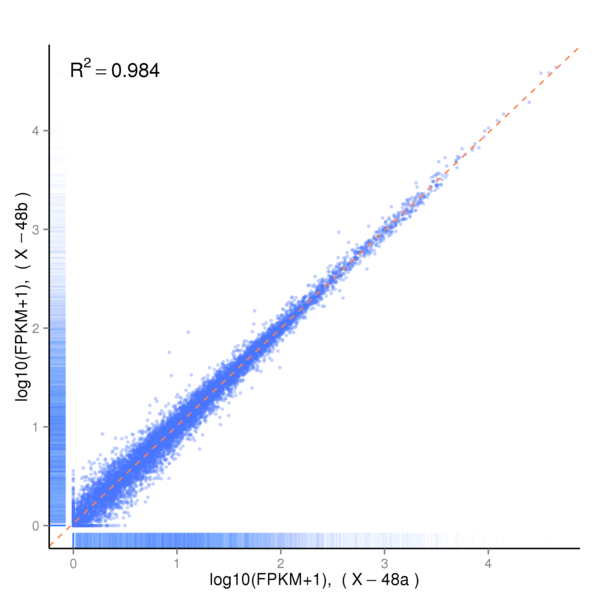


**Vd-X-48a vs Vd-X-48b**


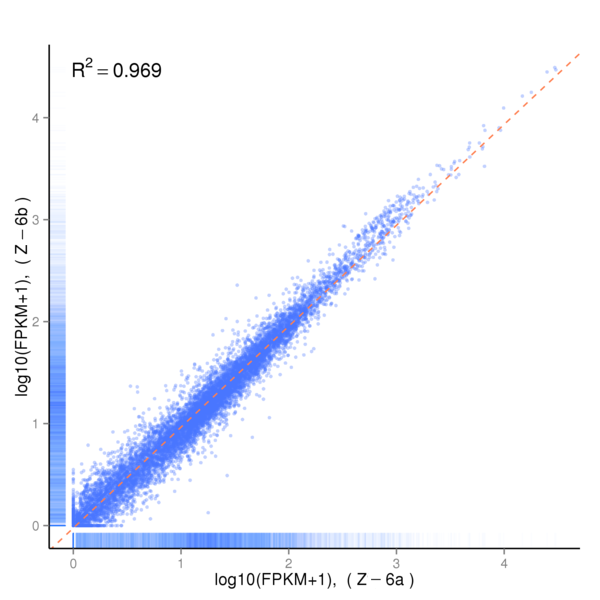


**Vd-Z-6a vs Vd-Z-6b**


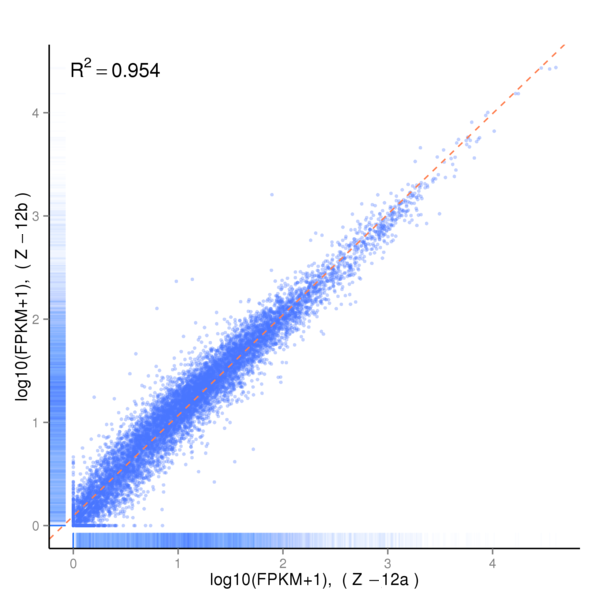


**Vd-Z-12a vs Vd-Z-12b**


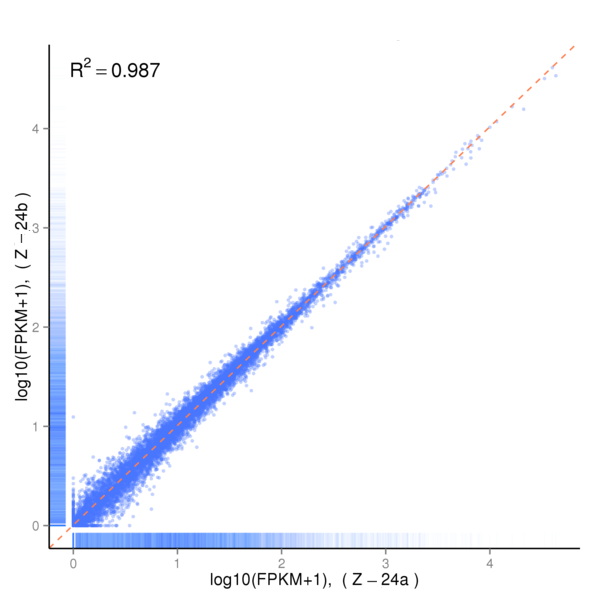


**Vd-Z-24a vs Vd-Z-24b**


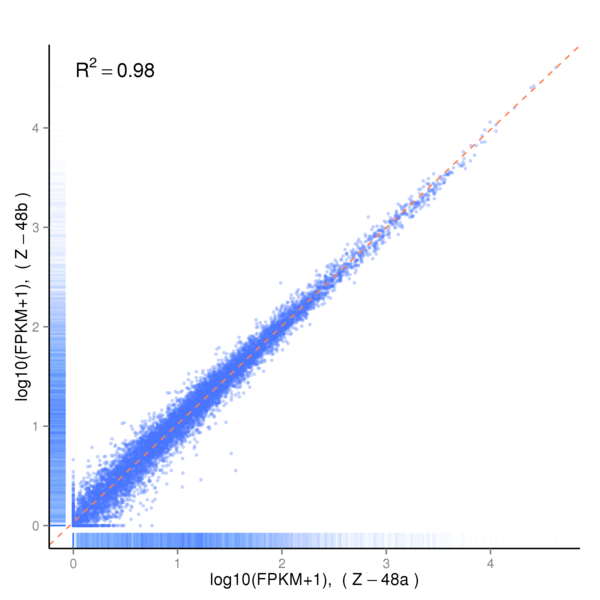


**Vd-Z-48a vs Vd-Z-48b**


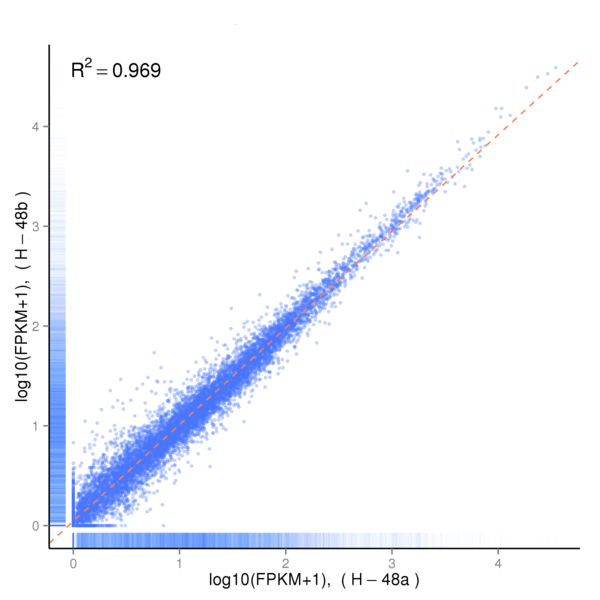

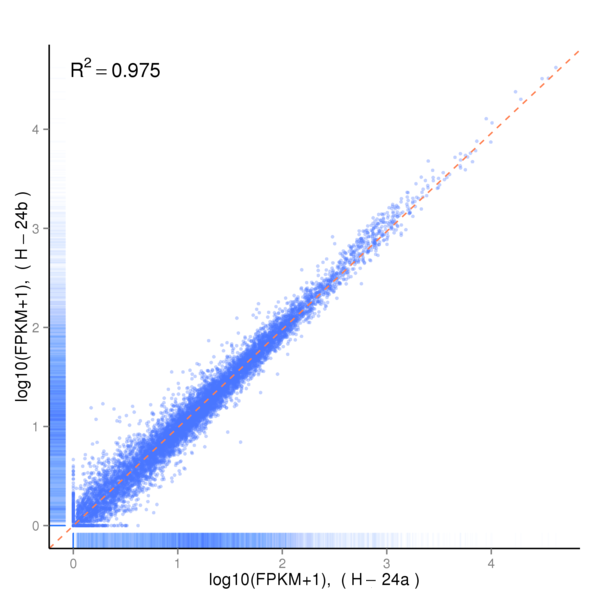


**Vd-H-24a vs Vd-H-24b**

**Vd-H-48a vs Vd-H-48b**


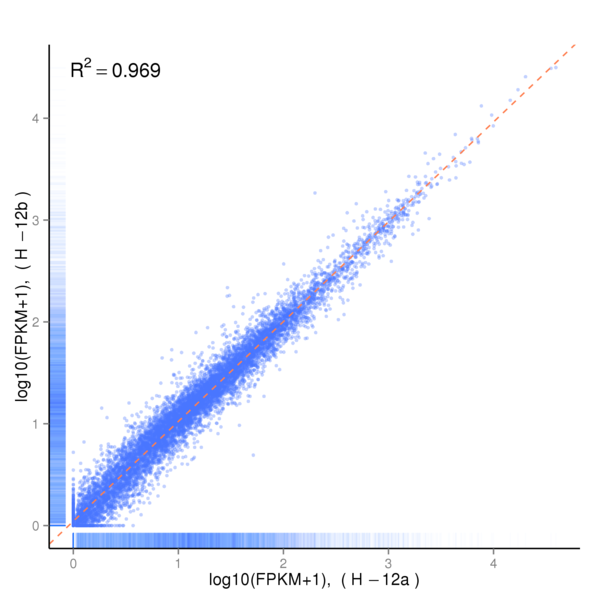

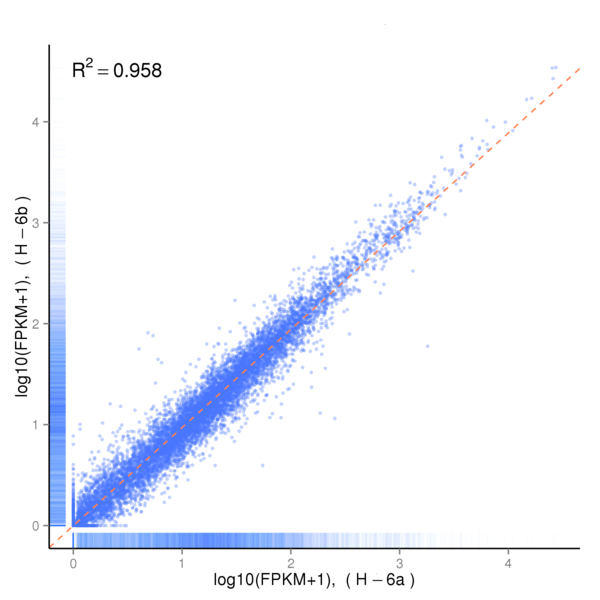


**Vd-H-6a vs Vd-H-6b**

**Vd-H-12a vs Vd-H-12b**


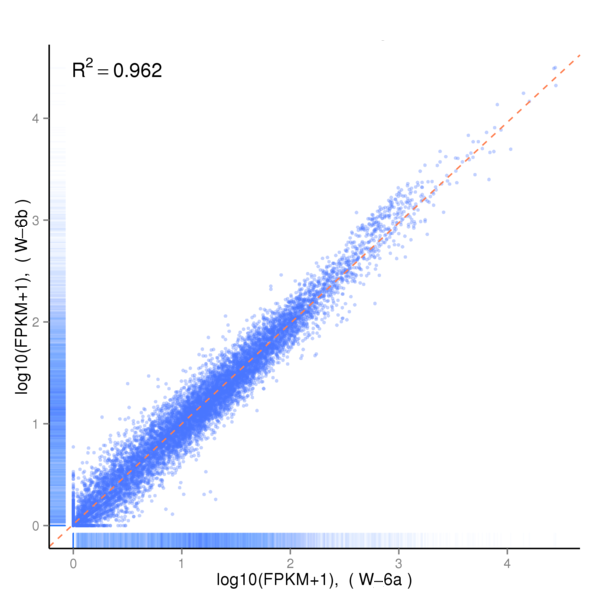


**Vd-W-6a vs Vd-W-6b**


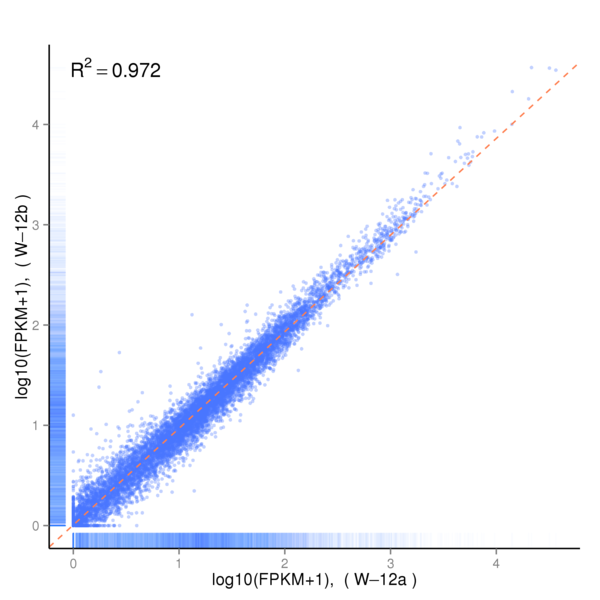


**Vd-W-12a vs Vd-W-12b**


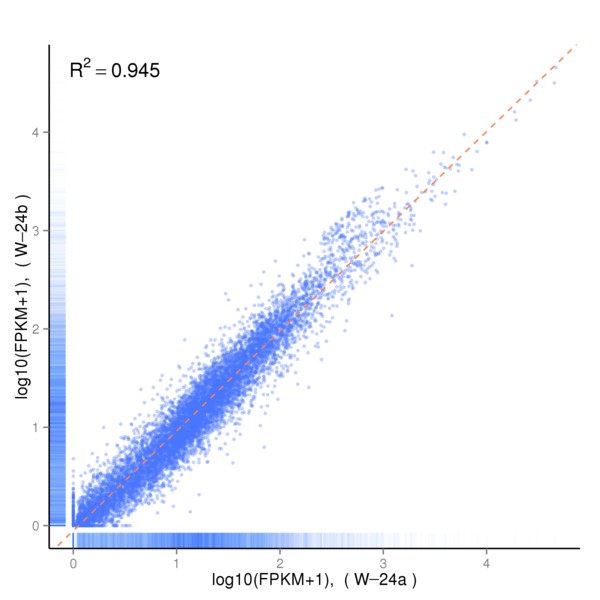


**Vd-W-24a vs Vd-W-24b**


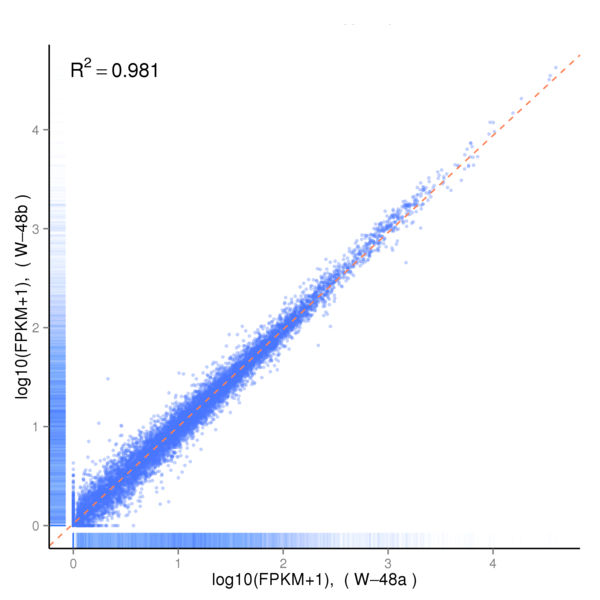


**Vd-W-48a vs Vd-W-48b**


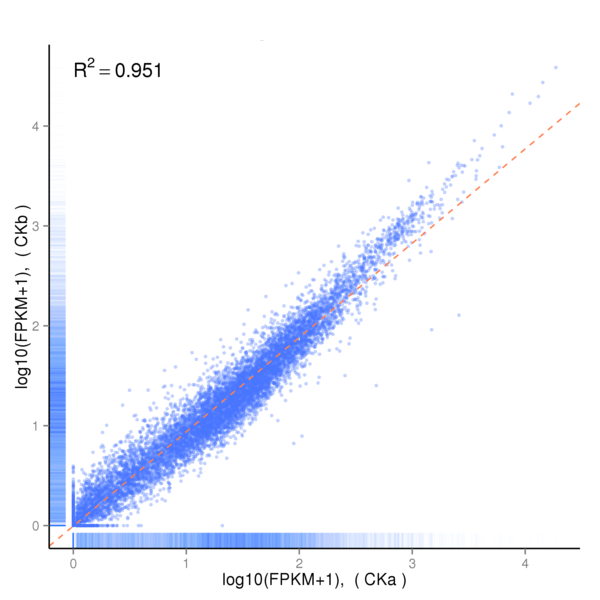


**CKa vs CKb(Vd-0a vs Vd-0b)**


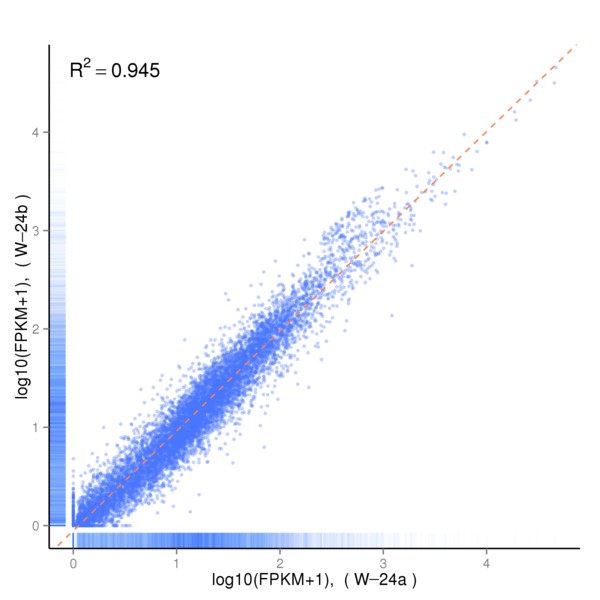


**Vd-W-24a vs Vd-W-24b**


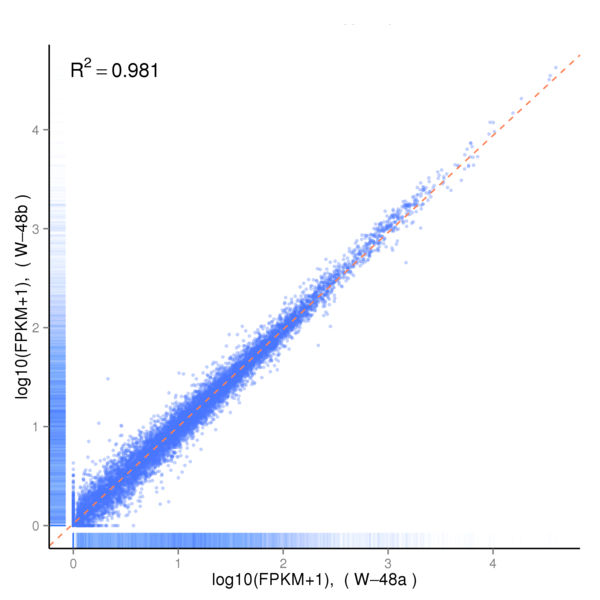


**Vd-W-48a vs Vd-W-48b**
