# Supplementary material for: Transcriptomic analysis of gene expression of Verticillium dahliae upon treatment of the cotton root exudates
Source: BMC Genomics. 2020 Feb 12;21:155. doi: 10.1186/s12864-020-6448-9 (PMC7017574; doi:10.1186/s12864-020-6448-9)
Supplement: Supplementary file 2 [file 12864_2020_6448_MOESM2_ESM.docx]

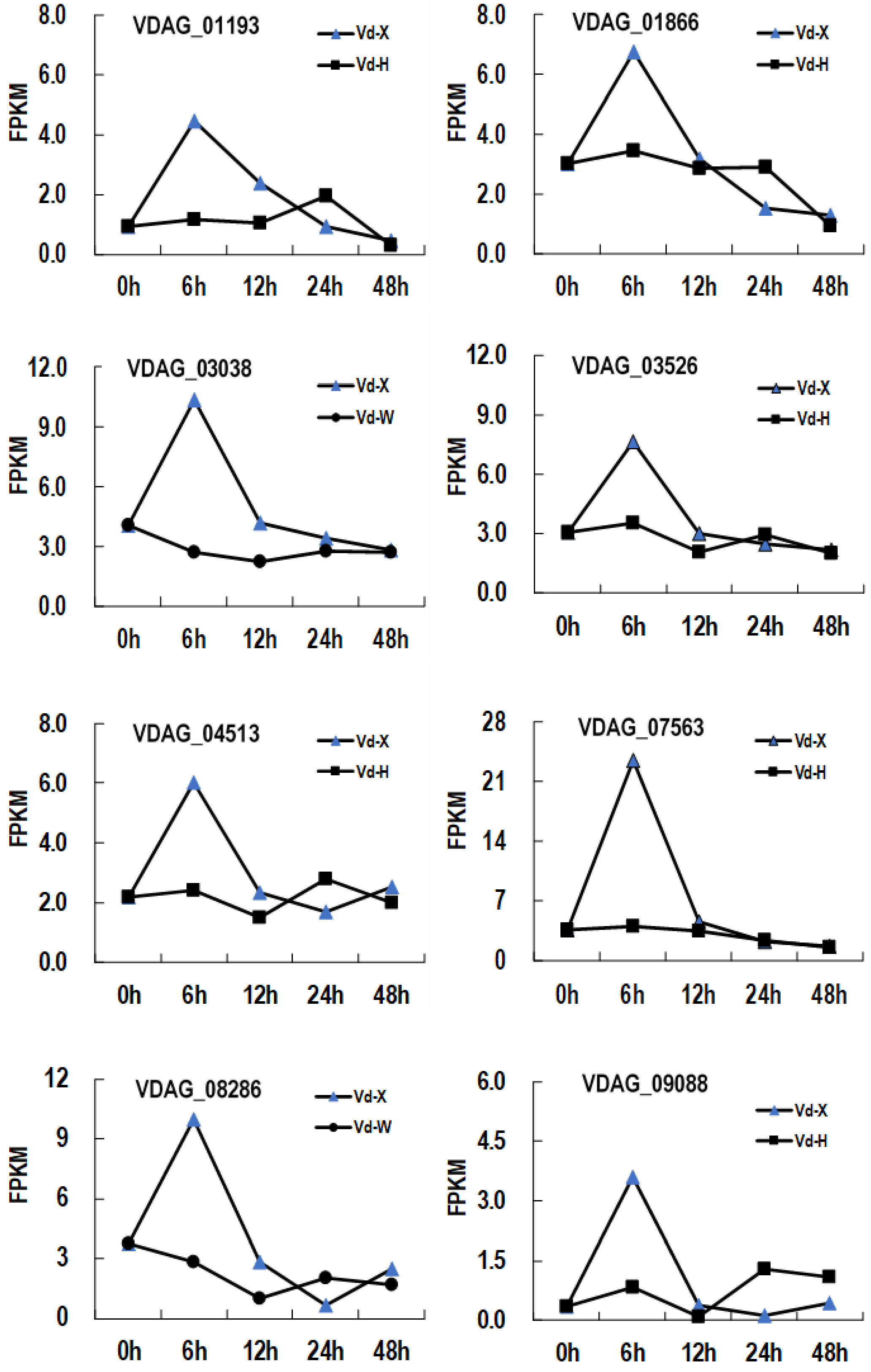

VDAG_01193
Vd-X
Vd-H
FPKM
8.0
6.0
4.0
2.0
0.0
0h
6h
12h
24h
48h
VDAG_01866
Vd-X
Vd-H
FPKM
8.0
6.0
4.0
2.0
0.0
0h
6h
12h
24h
48h
VDAG_03038
Vd-X
Vd-W
FPKM
12.0
9.0
6.0
3.0
0.0
0h
6h
12h
24h
48h
VDAG_03526
Vd-X
Vd-H
FPKM
12.0
9.0
6.0
3.0
0.0
0h
6h
12h
24h
48h
VDAG_04513
Vd-X
Vd-H
FPKM
8.0
6.0
4.0
2.0
0.0
0h
6h
12h
24h
48h
VDAG_07563
Vd-X
Vd-H
FPKM
28
21
14
7
0
0h
6h
12h
24h
48h
VDAG_08286
Vd-X
Vd-W
FPKM
12
9
6
3
0
0h
6h
12h
24h
48h
VDAG_09088
Vd-X
Vd-H
FPKM
6.0
4.5
3.0
1.5
0.0
0h
6h
12h
24h
48h
